# Supplementary material for: Effects of nutrition education and home gardening interventions on feto-maternal outcomes among pregnant women in Jimma Zone, Southwest Ethiopia: A cluster randomized controlled trial
Source: PLoS One. 2023 Oct 20;18(10):e0288150. doi: 10.1371/journal.pone.0288150 (PMC10588865; doi:10.1371/journal.pone.0288150)
Supplement: S3 File — (DOCX) [file pone.0288150.s003.docx]

Generalized estimating equation model predicting the effect of the intervention on hemoglobin level of pregnant women in Jimma Zone, Southwest Ethiopia, 2020

| **Variables** | | **Β** | **SE** | **P-value** | **95% CI** | |
| --- | --- | --- | --- | --- | --- | --- |
|  |  |  |  |  | **Lower** | **Upper** |
| **Hemoglobin level** | Intercept | 12.55 | 0.12 | < 0.001 | 12.30 | 12.80 |
|  | **Groups** |  |  |  |  |  |
|  | Husband | 0.15 | 0.17 | 0.37 | 0.19 | 0.50 |
|  | Peers | 0.03 | 0.18 | 0.85 | 0.32 | 0.38 |
|  | Control | Ref |  |  |  |  |
|  | Time | 0.44 | 0.10 | < 0.001 | 0.22 | 0.65 |
|  | Time*husband | 0.45 | 0.15 | 0.004 | 0.14 | 0.76 |
|  | Time* peer | 0.05 | 0.17 | 0.76 | 0.29 | 0.40 |
|  | **Maternal age** | -0.002 | 0.01 | 0.88 | - 0.003 | 0.02 |
|  | **Maternal education** |  |  |  |  |  |
|  | No formal education | 0.19 | 0.33 | 0.006 | 0.26 | 1.56 |
|  | Elementary school | 0.64 | 0.30 | 0.03 | 0.04 | 1.25 |
|  | Complete grade 8 | 1.05 | 0.35 | 0.003 | 0.36 | 1.73 |
|  | High school | 0.59 | 0.43 | 0.16 | -0.24 | 1.44 |
|  | Complete high school and above | Ref. |  |  |  |  |
|  | **Maternal occupation** |  |  |  |  |  |
|  | Merchant | 0,20 | 0.38 | 0.59 | -0.55 | 0.96 |
|  | Housewife | 0.26 | 0.36 | 0.46 | 0.45 | 0.98 |
|  | Government employee | 0.81 | 0.49 | 0.10 | -0.16 | 1.78 |
|  | Student | 0.24 | 0.62 | 0.69 | -0.97 | 1.46 |
|  | Daily laborers | Ref. |  |  |  |  |
|  | **Family size** |  |  |  |  |  |
|  | Less than five | 0.01 | 0.11 | 0.90 | 0.21 | 0.23 |
|  | Greater than five | Ref. |  |  |  |  |
|  | **Wealth index** |  |  |  |  |  |
|  | Rich | -0.72 | 0.63 | 0.25 | -1.96 | 0.51 |
|  | Medium | 0.22 | 0.12 | 0.06 | 0.47 | 3.43 |
|  | Poor | Ref. |  |  |  |  |
|  | **Alcohol consumption** |  |  |  |  |  |
|  | Yes | 0.78 | 024 | 0.001 | 0.31 | 1.25 |
|  | No | Ref. |  |  |  |  |
|  | **Khat chewing** |  |  |  |  |  |
|  | Yes | 0.05 | 0.15 | 0.70 | 0.35 | 0.14 |
|  | No | Ref. |  |  |  |  |
|  | **Districts** |  |  |  |  |  |
|  | Mainly coffee produce | -0.09 | 0.11 | 0.43 | -0.32 | 0.13 |
|  | Mainly grain producer | Ref. |  |  |  |  |
