# Supplementary material for: Effects of nutrition education and home gardening interventions on feto-maternal outcomes among pregnant women in Jimma Zone, Southwest Ethiopia: A cluster randomized controlled trial
Source: PLoS One. 2023 Oct 20;18(10):e0288150. doi: 10.1371/journal.pone.0288150 (PMC10588865; doi:10.1371/journal.pone.0288150)
Supplement: S5 File — (DOCX) [file pone.0288150.s005.docx]

Generalized estimating equation model predicting the effect of the intervention on minimum diet diversity of pregnant women in Jimma Zone, Southwest Ethiopia, 2020

| **Variables** | | **Β** | **SE** | **P-value** | **95% CI** | |
| --- | --- | --- | --- | --- | --- | --- |
|  |  |  |  |  | **Lower** | **Upper** |
| **MDD-W** | Intercept | 23.12 | 0.17 | <0.001 | 22.78 | 23.45 |
|  | **Groups** |  |  |  |  |  |
|  | Husband | 0.006 | 0.23 | 0.77 | 0.39 | 0.52 |
|  | Peer | 0.19 | 0.23 | 0.40 | 0.27 | 0.66 |
|  | Control | Ref |  |  |  |  |
|  | **Time** | 0.66 | 0.11 | <0.001 | 0.44 | 0.88 |
|  | Time*Husband | 0.16 | 0.16 | 0.31 | 0.15 | 0.45 |
|  | Time*Peer | 0.26 | 019 | 0.16 | 0.10 | 0.64 |
|  | **Maternal age** | 0.04 | 0.02 | 0.06 | -0.002 | 0.08 |
|  | **Maternal education** |  |  |  |  |  |
|  | No formal education | -1.22 | 0.62 | 0.05 | -2.45 | 0.004 |
|  | Elementary school | -1.36 | 0.61 | 0.02 | -2.58 | 0.16 |
|  | Complete grade 8 | -1.22 | 0.64 | 0.05 | -2.48 | 0.03 |
|  | High school | 0.80 | 0.70 | 0.25 | -2.17 | 0.66 |
|  | Complete high school and above | Ref. |  |  |  |  |
|  | **Maternal occupation** |  |  |  |  |  |
|  | Merchant | -1.87 | 0.61 | 0.002 | -3.08 | - 0.67 |
|  | Housewife | -1.77 | 0.59 | 0.003 | - 2.93 | - 0.8 |
|  | Government employee | -2.38 | 1.02 | 0.02 | - 4.39 | - 0.37 |
|  | Student | -2.08 | 0.87 | 0.01 | - 3.79 | - 0.36 |
|  | Daily laborers | Ref. |  |  |  |  |
|  | **Family size** |  |  |  |  |  |
|  | Less than five | 0.28 | 0.26 | 0.31 | -0.27 | 0.8 |
|  | Greater than five | Ref. |  |  |  |  |
|  | **Wealth index** |  |  |  |  |  |
|  | Rich | 0.72 | 0.59 | 0.22 | - 0.44 | 1.89 |
|  | Medium | 0.36 | 0.18 | 0.05 | 0.004 | 0.72 |
|  | Poor | Ref. |  |  |  |  |
|  | **Alcohol consumption** |  |  |  |  |  |
|  | Yes | 1.07 | 0.43 | 0.01 | 0.22 | 1.92 |
|  | No | Ref. |  |  |  |  |
|  | **Khat chewing** |  |  |  |  |  |
|  | Yes | - 0.05 | 0.20 | 0.81 | - 0.45 | 0.35 |
|  | No |  |  |  |  |  |
|  | **Districts** |  |  |  |  |  |
|  | Mainly coffee produce | 0.15 | 0.17 | 0.37 | - 0.18 | 0.49 |
|  | Mainly grain producer | Ref. |  |  |  |  |
|  | **Food insecurity** | - 0.06 | - 0.02 | 0.06 | - 0.11 | - 0.02 |
