# Supplementary material for: Effects of nutrition education and home gardening interventions on feto-maternal outcomes among pregnant women in Jimma Zone, Southwest Ethiopia: A cluster randomized controlled trial
Source: PLoS One. 2023 Oct 20;18(10):e0288150. doi: 10.1371/journal.pone.0288150 (PMC10588865; doi:10.1371/journal.pone.0288150)
Supplement: S6 File — (DOCX) [file pone.0288150.s006.docx]

Generalized estimating equation model predicting the effect of the intervention on dietary knowledge of pregnant women in Jimma Zone, Southwest Ethiopia, 2020

| **Variables** | | **Β** | **SE** | **P-value** | **95% CI** | |
| --- | --- | --- | --- | --- | --- | --- |
|  |  |  |  |  | **Lower** | **Upper** |
| **Dietary**  **Knowledge** | Intercept | 5.09 | 0.29 | < 0.001 | 4.51 | 5.67 |
|  | **Groups** |  |  |  |  |  |
|  | Husband | 0.009 | 0.42 | 0.98 | -0.84 | 0.83 |
|  | Peers | 0.19 | 0.41 | 0.63 | -0.61 | 1.00 |
|  | Control | Ref |  |  |  |  |
|  | **Time** | 11.75 | 0.53 | < 0.001 | 10.71 | 12.80 |
|  | Time*Husband | 9.75 | 0.81 | < 0.001 | 8.15 | 11.34 |
|  | Time*Peers | 1.57 | 0.72 | < 0.03 | 0.01 | 2.99 |
|  | **Maternal age** | 0.11 | 0.05 | 0.03 | 0.21 | 4.43 |
|  | **Maternal education** |  |  |  |  |  |
|  | No formal education | -1.15 | 0.64 | 0.07 | - 2.41 | 0.10 |
|  | Elementary school | -1.85 | 0.55 | 0.001 | -2.94 | -0.75 |
|  | Complete grade 8 | -0.93 | 1.00 | 0.34 | -2.89 | 1.01 |
|  | High school | -1.07 | 0.80 |  | -2.64 | 0.49 |
|  | Complete high school and above | Ref. |  |  |  |  |
|  | **Maternal occupation** |  |  |  |  |  |
|  | Merchant | 1.74 | 0.90 | 0.05 | -0.04 | 3.52 |
|  | Housewife | 1.24 | 0.84 | 0.14 | -0.42 | 2.90 |
|  | Government employee | 3.97 | 1.71 | 0.02 | 0.61 | 7.32 |
|  | Student | -0.66 | 0.99 | 0.50 | 1.29 | 0.44 |
|  | Daily laborers | Ref. |  |  |  |  |
|  | **Family size** |  |  |  |  |  |
|  | Less than five | -0.94 | -0.51 |  | -1.94 | 0.06 |
|  | Greater than five | Ref. |  |  |  |  |
|  | **Wealth index** |  |  |  |  |  |
|  | Rich | -1.58 | 0.79 |  | -3.14 | -0.02 |
|  | Medium | -0.39 | 0.33 |  | -1.05 | 0.25 |
|  | Poor | Ref. |  |  |  |  |
|  | **Alcohol consumption** |  |  |  |  |  |
|  | Yes | -0.91 | 0.81 |  | -2.51 | 0.68 |
|  | No | Ref. |  |  |  |  |
|  | **Khat chewing** |  |  |  |  |  |
|  | Yes | 0.07 | 0.37 |  | -0.67 | 0.81 |
|  | No | Ref. |  |  |  |  |
|  | **Districts** |  |  |  |  |  |
|  | Mainly coffee produce | -0.02 | 0.32 |  | -0.66 | 0.61 |
|  | Mainly grain producer | Ref. |  |  |  |  |
|  | **Food insecurity** | 0.01 | 0.04 |  | -0.08 | 0.10 |
