# Supplementary material for: Effects of nutrition education and home gardening interventions on feto-maternal outcomes among pregnant women in Jimma Zone, Southwest Ethiopia: A cluster randomized controlled trial
Source: PLoS One. 2023 Oct 20;18(10):e0288150. doi: 10.1371/journal.pone.0288150 (PMC10588865; doi:10.1371/journal.pone.0288150)
Supplement: S7 File — (DOCX) [file pone.0288150.s007.docx]

Generalized estimating equation model predicting the effect of the intervention on the dietary attitude of pregnant women in Jimma Zone, Southwest Ethiopia, 2020

| **Variables** | | **Β** | **SE** | **P-value** | **95% CI** | |
| --- | --- | --- | --- | --- | --- | --- |
|  |  |  |  |  | **Lower** | **Upper** |
| **Dietary**  **Attitude** | **Intercept** | 22.72 | 0.22 | < 0.001 | 22.27 | 23.17 |
|  | **Groups** |  |  |  |  |  |
|  | Husband | -0.17 | 0.30 | 0.57 | - 0.75 | 0.41 |
|  | Peer | 0.04 | 0.31 | 0.88 | - 0.56 | 0.65 |
|  | Control | Ref |  |  |  |  |
|  | **Time** | 1.73 | 0.26 | < 0.001 | 1.21 | 1.25 |
|  | Time*husband | 1.92 | 0.41 | < 0.001 | 1.11 | 2.74 |
|  | Time*Peer | 0.16 | 0.38 | 0.67 | - 0.59 | 0.91 |
|  | **Maternal age** | -0.02 | 0.02 | 0.32 | -0.06 | 0.02 |
|  | **Maternal education** |  |  |  |  |  |
|  | No formal education | 0.53 | 0.57 | 0.35 | -0.59 | 1.66 |
|  | Elementary school | 0.49 | 0.55 | 0.36 | -0.58 | 1.57 |
|  | Complete grade 8 | 0.55 | 0.60 | 0.36 | -0.63 | 1.73 |
|  | High school | -0.85 | 0.67 | 0.20 | -2.18 | 0.47 |
|  | Complete high school and above | Ref. |  |  |  |  |
|  | **Maternal occupation** |  |  |  |  |  |
|  | Merchant | -0.61 | 0.69 | 0.37 | -1.98 | 0.74 |
|  | Housewife | -0.44 | 0.69 | 0.52 | -1.80 | 0.91 |
|  | Government employee | -0.26 | 0.88 | 0.77 | -2.00 | 1.48 |
|  | Student | -0.99 | 1.05 | 0.34 | -3.05 | 1.07 |
|  | Daily laborers | Ref. |  |  |  |  |
|  | **Family size** |  |  |  |  |  |
|  | Less than five | 0.06 | 0.28 | 0.82 | -0.50 | 0.62 |
|  | Greater than five | Ref. |  |  |  |  |
|  | **Wealth index** |  |  |  |  |  |
|  | Rich | 0.74 | 0.63 | 0.25 | -0.50 | 1.99 |
|  | Medium | -0.15 | 0.21 | 0.46 | -0.56 | 0.26 |
|  | Poor | Ref. |  |  |  |  |
|  | **Alcohol consumption** |  |  |  |  |  |
|  | Yes | 1.89 | 0.95 | 0.04 | 0.01 | 3.77 |
|  | No | Ref. |  |  |  |  |
|  | **Khat chewing** |  |  |  |  |  |
|  | Yes | 0.67 | 0.24 | 0.005 | 0.20 | 1.15 |
|  | No |  |  |  |  |  |
|  | **Districts** |  |  |  |  |  |
|  | Mainly coffee produce | 0.01 | 0.20 | 0.94 | -0.39 | 0.42 |
|  | Mainly grain producer | Ref. |  |  |  |  |
|  | **Food insecurity** | 0.04 | 0.03 | 0.17 | -0.01 | 0.10 |
