# Supplementary material for: Effects of nutrition education and home gardening interventions on feto-maternal outcomes among pregnant women in Jimma Zone, Southwest Ethiopia: A cluster randomized controlled trial
Source: PLoS One. 2023 Oct 20;18(10):e0288150. doi: 10.1371/journal.pone.0288150 (PMC10588865; doi:10.1371/journal.pone.0288150)
Supplement: S8 File — (DOCX) [file pone.0288150.s008.docx]

Generalized estimating equation model predicting the effect of the intervention on the dietary practice of pregnant women in Jimma Zone, Southwest Ethiopia, 2020

| **Variables** | | **Β** | **SE** | **P-value** | **95% CI** | |
| --- | --- | --- | --- | --- | --- | --- |
|  |  |  |  |  | **Lower** | **Upper** |
| **Dietary**  **Practice** | **Intercept** | 2.08 | 0.09 | < 0.001 | 1.90 | 2.27 |
|  | **Groups** |  |  |  |  |  |
|  | Husband | 0.06 | 0.13 | 0.64 | - 0.20 | 0.32 |
|  | Peer | 0.16 | 0.12 | 0.20 | - 0.08 | 0.41 |
|  | Control | Ref |  |  |  |  |
|  | **Time** | 2.69 | 0.10 | < 0.001 | 2.50 | 2.89 |
|  | Time*husband | 0.69 | 0.16 | < 0.001 | 0.37 | 1.01 |
|  | Time*Peer | 0.24 | 0.14 | 0.10 | - 0.52 | 0.04 |
|  | **Maternal age** | -0.01 | 0.009 | 0.22 | -0.02 | 0.007 |
|  | **Maternal education** |  |  |  |  |  |
|  | No formal education | -0.14 | 0.23 | 0.52 | -0.60 | 0.30 |
|  | Elementary school | -0.12 | 0.22 | 0.57 | -0.56 | 0.31 |
|  | Complete grade 8 | 0.10 | 0.23 | 0.64 | -0.35 | 0.56 |
|  | High school | 0.05 | 0.23 | 0.82 | -0.40 | 0.51 |
|  | Complete high school and above | Ref. |  |  |  |  |
|  | **Maternal occupation** |  |  |  |  |  |
|  | Merchant | -0.14 | 0.18 | 0.46 | -0.51 | 0.22 |
|  | Housewife | -0.18 | 0.18 | 0.30 | -0.55 | 0.17 |
|  | Government employee | 0.46 | 0.33 | 0.16 | -0.19 | 1.13 |
|  | Student | 0.21 | 0.26 | 0.42 | -0.30 | 0.72 |
|  | Daily laborers | Ref. |  |  |  |  |
|  | **Family size** |  |  |  |  |  |
|  | Less than five | -0.13 | 0.11 | 0.9 | -0.23 | 0.21 |
|  | Greater than five | Ref. |  |  |  |  |
|  | **Wealth index** |  |  |  |  |  |
|  | Rich | -0.08 | 0.22 | 0.35 | -0.51 | 0.35 |
|  | Medium | -0.003 | 0.07 | 0.97 | -0.15 | 0.14 |
|  | Poor | Ref. |  |  |  |  |
|  | **Alcohol consumption** |  |  |  |  |  |
|  | Yes | 0.16 | 0.16 | 0.32 | -0.16 | 0.48 |
|  | No | Ref. |  |  |  |  |
|  | **Khat chewing** |  |  |  |  |  |
|  | Yes | 0.03 | 0.09 | 0.75 | -0.16 | 0.22 |
|  | No |  |  |  |  |  |
|  | **Districts** |  |  |  |  |  |
|  | Mainly coffee produce | 0.02 | 0.07 | 0.77 | -0.12 | 0.16 |
|  | Mainly grain producer |  |  |  |  |  |
|  | **Food insecurity** | -0.004 | 0.11 | 0.76 | -0.02 | 0.01 |
