# Supplementary material for: Effects of nutrition education and home gardening interventions on feto-maternal outcomes among pregnant women in Jimma Zone, Southwest Ethiopia: A cluster randomized controlled trial
Source: PLoS One. 2023 Oct 20;18(10):e0288150. doi: 10.1371/journal.pone.0288150 (PMC10588865; doi:10.1371/journal.pone.0288150)
Supplement: S9 File — (DOCX) [file pone.0288150.s009.docx]

**Effects of nutrition education and home gardening interventions on feto-maternal outcomes among pregnant women in Jimma Zone, Southwest Ethiopia**

**Principal Investigator:**

**Melesse Niguse Kuma, MSc, Ph.D. fellow**

Department of Nutrition and Dietetics, Jimma University

[meleseniguse@gmail.com](mailto:ggoba@uic.edu)

**Co- Investigators:**

**Tefera Belachew, MD,** **MSc, Ph.D.**

Professor of Human Nutrition Department of Nutrition and Dietetics, Jimma University

Director of post-Graduate Studies, Jimma University

teferabelachew2@gmail.com

**Dessalegn Tamiru, MSc, Ph.D.**

Associate professor, Department of Nutrition and Dietetics, Jimma University

Head Department of Nutrition and Dietetics, Jimma University

dessalegn97@gmail.com

**Jimma Ethiopia**,

February 2019

# Abstract

**Background**: Adequate maternal nutrition before and during pregnancy plays a key role in the health outcomes of the mother and neonate. Nutrition-sensitive programs are important in the prevention of maternal malnutrition, especially in low-income countries like Ethiopia. Home gardens are a part of the nutrition-sensitive agricultural programs practiced in many developing countries. In addition to this, home gardens are a time-tested local strategy that is widely adopted and practiced in various circumstances by local communities with limited resources and institutional support. However, there were inconsistent reports on the improvement of nutritional status and feto-maternal outcomes of pregnancy. In addition to this, to our knowledge, there is no report from the study area on the synergistic effect of home gardening and nutritional education on pregnancy outcomes.

**Objective:** the objective of this study is to assess the combined effect of nutritional education and home gardening interventions on feto-maternal outcomes among pregnant women of Jimma zone, southwest Ethiopia.

**Methodology**: The study design will be a cluster randomized controlled trial done on pregnant mothers from August 2020 to December 2019. Two districts will be selected randomly one from each agro-ecological area of the zone (predominantly coffee growing and grain growing). Then, non-adjoining Kebeles/clusters will be identified and marked on the map. Finally, by using a random sampling method clusters from both districts will be assigned to the intervention or control group. The sample size will be determined by G-power 3.1. The number of pregnant mothers to be involved in the study from both woreda will be made by the population proportion of each cluster. The statistical analysis method to be used will be descriptive, Oneway analysis of Variance, and the Generalized estimating equation model will be used.

**Resul**t: The result of this study will be described and displayed in tables and figures. The total budget needed for the study will be 603,130 ETB. The conclusions and recommendations will be drowning based on the result.

Keywords: Pregnancy, Home gardening, Pregnancy outcomes, Nutritional education.

# ACKNOWLEDGEMENT

I would like to express my deepest appreciation and gratitude to my advisors Professor Dr. Tefera Belachew and Dr. Dessalegn Tamiru for their valuable comments and guidance in designing and writing this proposal. Also, my thanks go to Jimma University, Faculty of public health, department of Population and family health for giving me this golden opportunity to learn and enabling me to prepare this proposal. I would like to extend my thanks to my family, friends, and those who encourage, supported, and help me by giving information during the preparation.

Contents

[Abstract 2](#_Toc137962025)

[ACKNOWLEDGEMENT 3](#_Toc137962026)

[List of tables and figures 6](#_Toc137962027)

[Abbreviations and Acronyms 7](#_Toc137962028)

[CHAPTER ONE: INTRODUCTION 9](#_Toc137962029)

[1.1. BACKGROUND OF THE PROBLEM 9](#_Toc137962030)

[1.2. Statement of the problem 11](#_Toc137962031)

[*1.*3. Significance of the study 13](#_Toc137962032)

[1.3. Research hypothesis 17](#_Toc137962033)

[CHAPTER Two: OBJECTIVES 17](#_Toc137962034)

[2.1. General objective 17](#_Toc137962035)

[2.2. Specific objective 17](#_Toc137962036)

[CHAPTER THREE: METHODS AND MATERIALS 18](#_Toc137962037)

[3.1. The study settings 18](#_Toc137962038)

[3.2. Source population 18](#_Toc137962039)

[3.2.1*.* Study population 18](#_Toc137962040)

[3.2.2. Study Units 18](#_Toc137962041)

[3.2.3. Inclusion criteria 19](#_Toc137962042)

[3.2.4. Exclusion criteria 19](#_Toc137962043)

[3.3. Study design 19](#_Toc137962044)

[3.4. Sample Size Determinations 19](#_Toc137962045)

[3.5. Sampling procedures 19](#_Toc137962046)

[3.6. Study Variables 23](#_Toc137962047)

[3.6.1. Dependent variables 23](#_Toc137962048)

[3.6.2. Independent variables 23](#_Toc137962049)

[3.7. Timing and outputs of fieldwork 23](#_Toc137962050)

[3.7.1. Preparatory phase 23](#_Toc137962051)

[3.7.2. Intervention phase 23](#_Toc137962052)

[3.7.3. Monitoring and Evaluation Phase 25](#_Toc137962053)

[3.7.4. Compliance 26](#_Toc137962054)

[3.8. Data collection and instruments 29](#_Toc137962055)

[3.9. Data Quality Control 29](#_Toc137962056)

[3.10. Pretest 29](#_Toc137962057)

[3.11. Dietary intake assessment 30](#_Toc137962058)

[3.12. Anthropometric measurements 30](#_Toc137962059)

[3.13. Hemoglobin level Determination 30](#_Toc137962060)

[3.14. Statistical analysis 30](#_Toc137962061)

[3.15. Ethical Considerations. 31](#_Toc137962062)

[CHAPTER Four: Budget 32](#_Toc137962063)

[4.1. Budget Summary 33](#_Toc137962064)

[CHAPTER Five: Work plan 34](#_Toc137962065)

[CHAPTER SEVEN: Reference 36](#_Toc137962066)

# List of tables and figures

Figure1. The conceptual framework of the combined effect of home gardening and nutritional social behavioral change communication on feto-maternal outcomes. 16

Figure 2. Map of the study area with selected study clusters (kebeles) of both districts (Seka and Mana) source: Generated using ArcGIS software version 10.3 from ETHIO-GIS, 2014 database. 21

Figure 3. Shows that the sampling procedure for the effect of home gardening and social behavioral change communication on feto-maternal outcomes in Jimma zone, South West Ethiopia, 2019. 22

Table 2: Shows the summary of main interventional activities protocol of the effect of home gardening and social behavioral change communications on fetal outcomes in Jimma Zone, Southwest Ethiopia, 2019. 27

Table 4. Shows the budget summary of the effect of Home gardening and Social Behavioral change communication on feto-maternal outcomes in Jimma Zone, South West Ethiopia, 2019. 33

Table 5.Shows the work plan of the effect of Home gardening and Social Behavioral Change communication on feto-maternal outcomes in Jimma Zone, South West Ethiopia, 2019. 34

# Abbreviations and Acronyms

AHEI-P Alternate health eating index for pregnancy

ANC Antenatal care

AOR Adjusted Odds Ratio

BMI Body Mass Index

CHF Congestive heart failure

CHO Carbohydrate

CI Confidence Interval

CSA Central Statistical Authority

DA Development agent

DHS Demographic and Health Survey

DRI Dietary Reference Intakes

ENGINE Empowering New Generation to improve nutrition and Economic Opportunities

FAO Food and Agriculture Organization

FGD Focus group discussions

FVS Food variety score

GA Gestational age

GDM Gestational diabetes mellitus

GHI Global Health index

Hb Hemoglobin

HDP Hypertensive disorder of pregnancy

HDDS Household Dietary Diversity Score

HEW Health extension worker

IFA Iron Folic Acid

IOM Institute of Medicine

Kg Kilogram

LBW Low Birth Weight

LMIC Low- and Middle-Income Countries

MAR Mean adequacy ratio

MDD-W Minimum Dietary Diversity for Women of Reproductive Age

MDD Minimum Dietary Diversity

MUAC Mid-Upper Arm Circumference

NAR Nutrient adequacy ratio

NNP National Nutritional program

PTB Pre-tem Birth

SBCC Social and behavioral change communication

SNNP South nation nationalities and peoples

SPPS Statistical Package for Social Sciences

STI Sexually transmitted infection

UNICEF United Nations International Children's Emergency

WASH Water, sanitation, and hygiene

WDDS Women’s Dietary Diversity Score

WHO World Health Organization

Wt Weight

# CHAPTER ONE: INTRODUCTION

## BACKGROUND OF THE PROBLEM

Adequate maternal nutrition before and during pregnancy will play a pivotal role in the health outcomes of the mother and neonate. (De Silva Lopes K et al., 2017, Ramalho AA et al., 2017, Christian. 2018,). Accordingly, the nutritional status of the mother at conception influences how nutrients will be portioned between the mother and fetus (Janet C et al., 2003). Thus, poor maternal nutritional status indicators during pregnancy like MUAC less than 21 cm and anemia were important predictors of adverse neonatal outcomes (Hutagalung, 2017, Joshua et al., 2018).

For insistence, low birth weights, stillbirths, and preterm births were among the most common ones related to inadequate nutritional status of the mother (Ota et al., 2015, Hutagalung, et al 2017).

Maternal undernutrition during pregnancy also contributes to fetal growth restriction, which increases the risk of neonatal deaths and, for survivors, of stunting by 2 years of age (Black et al., 2013, Solomons et al., 2015). Similar studies also reported that early onset of growth delay and prematurity significantly increases the risk for a neurological abnormality, motor, and cognitive delay *(Stefania Longo et al., 2013).* Evidence from famine studies revealed that maternal nutritional deprivation during mid and late pregnancy was associated with significantly reduced fetal birth weight. While, if it is during early pregnancy, there will be a decline in the cognitive ability of the fetus in later life (Schulz et al., 2010, de Rooji S et al., 2010). Other similar studies also show that inadequate maternal nutrition during pregnancy will determine (“programmed”) the later age bone mineral metabolism and development of heart failure ( Barker D J. Pet al., 2010, George M et al., 2014).

Food insecurity during pregnancy was not associated only with adverse child outcomes but, also has an association with many maternal complications like maternal anemia, depression/anxiety disorders in pregnancy, gestational complications (diabetes, hypertension, obesity, and dyslipidemia), low gestational weight gain which are again the determinant factors for poor fetal-outcomes (Stefania Longo et al., 2013, Schoenaker et al., 2014, (Ramalho AA et al., 2017,).

Many identified factors make a woman vulnerable to malnutrition during pregnancy. For example, inadequate dietary intake, lack of availability of food, inequitable distribution of food within the same household, lack of knowledge about the importance of dietary diversity, and frequent occurrence of infectious diseases are among the common factors (*Darnton-Hill, 2012)*. In low and middle-income countries poor dietary intake is a major contributor (Ruel MT et al., 2013).

Globally, malnutrition of all its forms affects almost every country in the world and is a serious public health problem (Development Initiatives, 2017). Maternal undernutrition is one of these malnutrition forms which accounts for about 800 000 neonatal deaths annually (Black et al., 2013). Of child malnutrition forms, Stunting begins while in utero and continues for at least the first 2 years of post‐natal life and will be left with many sequels (Solomons NW et al., 2015). Its global prevalence in 2010 was 171 million(de onis et al., 2013). Even though this prevalence in children younger than 5 years has decreased during the past two decades, it is still high in South Asia and sub-Saharan Africa than elsewhere (Black *et al.*, 2013), Development Initiatives, 2017). Ethiopia is one of the very few African countries that can substantially decrease the stunting rate (from 57% in 2000 to 38% in 2016)(Central Statistical Agency (CSA) [Ethiopia] and ICF., 2016) but, the rate is still much below the international target.

Accordingly, different targets were set globally and locally to tackle this problem. To mention, 2012 World health organization (WHO) set a global target of reducing the number of under-five stunting by 40% (de onis M, et al, 2013), reducing and then maintaining levels of childhood wasting to below 5% by the year 2025 (WHO/UNICEF/WFP, 2014).

Malnutrition is the result of the complex array of casual and contextual factors which will require a multifaceted and multi-disciplinary approach suggested as a solution (de Onis M et al., 2016). Therefore, the 2013 Lancet series report suggests that nutrition-sensitive programs were important in the prevention of maternal malnutrition, especially in low-income countries, and also can help in scaling up nutrition-specific interventions, thereby creating a stimulating environment in which young children can grow and develop to their full potential(Ruel MT et al., 2013).

Home gardening is one the useful nutrition-sensitive food-based strategies to promote better-balanced diets among poor rural households that have access to a small plot of land and are willing to engage in gardening(Schreinemachers et al, 2016). A small-scale home-based food production program aimed at increasing access to nutritious foods has been found to support livelihoods and food security and proposed as a potentially useful platform for delivering nutrition-specific interventions targeted to women and young children (Black *et al.*, 2013). The effects of home gardening were often greater when combined with nutrition education intervention (Osei A et al., 2017). For instance, in Ethiopia the ENGINE (Empowering New Generation to improve nutrition and Economic Opportunities) project implemented nutrition-sensitive interventional activities to improve child malnutrition in different regions of selected 116 districts, through a multisectoral approach from September 2011–September 2016 was came up with impressive results. Stunting in children 3-36 months reduced by 20% in Amhara, 14% in SNNPR, and 12% in Oromia region. Also, the proportion of children meeting the minimum dietary diversity standards were more than double (Save the Children, 2016).

## Statement of the problem

Maternal malnutrition during pregnancy can cause irreversible damage to the newborn brain development and physical growth (Ruel MT et al., 2013., Black M M et al., 2015, de Onis M et al, 2016). This will lead to diminishing school performance capacity of the child, vulnerability to infection, and lost lifetime earning potential(Black M M et al., 2017). Similarly, maternal nutrient depletion may contribute to an increased incidence of preterm births and fetal growth retardation in addition to the risk of maternal mortality and morbidity(Janet C et al., 2003). Also, it can alter maternal hormones and increase the likelihood of prenatal complications such as gestational diabetes mellitus (GDM), preeclampsia, and intrauterine growth restriction*(Widen et ai., 2010, Al-Jemil N et al., 2017)*.

(Bhutta A Z, et al 2013) suggested that agricultural interventions were better in alleviating maternal malnutrition and breaking its intergenerational cycle through six identified pathways : (1) food access from own-production; (2) income from the sale of commodities produced; (3) food prices from changes in supply and demand; (4) women's social status and empowerment through increased access to and control over resources; (5) women's time through participation in agriculture (6) women's health and nutrition through engagement in agriculture.

Evidence shows that home gardens are a part of the agriculture and food production systems in many developing countries and are widely used as a remedy to alleviate hunger and malnutrition in the face of a global food crisis(Johnson-welch *et al.*, 2000).In addition to this, home gardens are a time-tested local strategy that is widely adopted and practiced in various circumstances by local communities with limited resources and institutional support(Schreinemachers et al, 2016).

Maternal undernutrition is prevalent, especially in low and middle-income countries (LMICs), with approximately 20% of women in Asia and 10% of women in Africa having low body mass index (BMI) (less than (<) 18·5 kg/m² in adult women(Black *et al.*, 2013). The UNICEF, WHO, and World Bank Group of 2016 jointly reported on fewer than five children showing that globally there were about 52 million wasted and 155 million stunted children. On the other hand, there were 41 million overweight children *(UNICEF, WHO, and World Bank Group, 2017*). The prevalence of stunting in Africa is increasing in contrast to the other world except in a few countries like; Ethiopia, Ghana, and Mauritania(*de onis M et al., 2013)*.

In Ethiopia according to the recent (DHS 2016) report, twenty-two percent of women of reproductive age were undernourished, leaving their children predisposed to low birth weight, short stature, low resistance to infections, and high risk of disease and death. Of the total childbearing-age female deaths, 25 percent were related to pregnancy or childbearing. The prevalence of stunting and acute malnutrition (wasting or low weight-for-height)in Ethiopia has decreased over the past decade but remains high, with 38 percent(5.8 million) of children under 5 years stunted and 10 percent wasted*. (Central Statistical Agency (CSA) [Ethiopia] and ICF., 2016). But* the prevalence of anemia in childbearing-age females was increasing from 2011 (17% to 24%) in 2016. Even though, significant steps the world has taken towards improving nutrition and associated health burdens over recent decades, the 2017 year Global Nutrition Report indicated that still nutrition is a universal large-scale problem(Development Initiatives., 2017).

In the same way, the government of Ethiopia also recognized nutritional problems as a major public health issue and made global and regional commitments to alleviate nutritional problems(FDRE, 2016) like; the July 2003 Maputo Declaration of comprehensive Africa Agriculture development program, Scaling Up Nutrition (SUN) Movement 2013, the global nutrition for growth compact made at the 2013 nutrition for growth summit in London, the African Union’s Malabo Declaration on accelerated agricultural growth and transformation for shared prosperity and improved livelihoods.

Also have made national commitments and strategies for instance; the five years national nutrition strategy of (2008), growth and transformation plan II (GTP), the revised second national nutrition program (NNP II) (2016–2020), and seqota Declaration (2015) are some of them. The Seqota Declaration aims to end hunger (zero stunting) and undernutrition by 2030 through nutrition-sensitive and nutrition-specific programs *(USAID, 2018),*(FDRE, 2016b)*(Federal Ministry of Health of Ethiopia, 2008)*. Despite these efforts still malnutrition is a major public health problem in the country.

## *1.*3. Significance of the study

It is well understood that maternal undernutrition during pregnancy can cause irreversible damage to the newborn's brain development and physical growth (Ruel et al., 2013., Black et al., 2015, de Onis et al, 2016). Thus, it would lead to the child's diminished school performance capacity, vulnerability to infection, and loss of lifetime earning potential (Black et al., 2017). In addition to this, maternal nutrient depletion during pregnancy may contribute to an increased incidence of preterm births and low birth weight, in addition to maternal mortality and morbidity risks (Janet et al., 2003).

Furthermore, it can alter maternal hormones and increase the likelihood of prenatal complications such as gestational diabetes mellitus (GDM), preeclampsia, and intrauterine growth restriction (Widen et al., 2010, Al-Jemil et al., 2017). Globally, for three consecutive years, the prevalence of undernourished people, i.e., those facing chronic food deprivation, has increased to nearly 821 million in 2017, from around 804 million in 2016. Africa continues to have the highest, affecting nearly 21% of the population (more than 256 million people) (FAO 2018).

Overall, stunting in children has decreased by 9 percent, from 165.2 million in 2012 to 150.8 million in 2017 (UNICEF, WHO, and World Bank Group, 2017, (FAO 2018). Conversely, anemia among women of reproductive age is not improving (30.3 percent in 2012 to 32.8 percent in 2016) with no region showing a decline. However, one in three women of reproductive age globally is still affected by anemia, with significant health and development consequences for both women and their children(FAO 2018). Therefore, the prevalence of malnutrition is still unacceptably high and the road to reaching the 2030 SDG target is still long.

Nowadays, as the world embarks on the final decade towards 2030, the recent UNICEF report indicates that at least one in three children is not growing well because of malnutrition, and at least two in three are not fed the minimum diet they need to grow, develop and learn to their full potential (UNICEF 2020). Likewise, new forces like globalization, urbanization, inequities, environmental crises, health epidemics, and humanitarian emergencies drive back the nutritional situation of feto-maternal and pose critical challenges to feeding them sustainably (Seferidi, Hone et al. 2022).

To avert this childhood malnutrition and improve other maternal and child health indicators, attention is being given to nutrition-specific and nutrition-sensitive interventions (Khalid, Gill et al. 2019). Nutrition-specific interventions refer to interventions that address the immediate determinants (Bhutta, Das et al. 2013). While nutrition-sensitive interventions influence the underlying determinants of nutrition and create a stimulating environment in which young children can grow and develop to their full potential (Ruel, Alderman et al. 2013). To mention some (Bhutta, et al 2013) suggested that nutritional sensitive agricultural interventions were better in alleviating maternal malnutrition and breaking the intergenerational cycle of malnutrition through six identified pathways : (1) food access from own production; (2) income from the sale of commodities produced; (3) food prices from changes in supply and demand; (4) women's social status and empowerment through increased access to and control over resources; (5) women's time through participation in agriculture (6) women's health and nutrition through engagement in agriculture.

Likewise, others reported that the home garden, one of the agricultural interventions that use time-tested local strategies that are widely adopted and practiced in various circumstances by local communities with limited resources, is effective in the prevention of feto-maternal malnutrition (Schreinemachers et al, 2016). On the other hand, a combination of agricultural interventions with social and behavioral change communications (nutrition-specific intervention) is more optimized for the alleviation of malnutrition as reported (Kennedy, Stickland et al. 2018).

Even though, significant steps the world has taken towards improving nutrition and associated health burdens over decades, the 2017 year Global Nutrition Report indicated that still nutrition is a universal large-scale problem (Development Initiatives., 2017).

In Ethiopia according to the (DHS 2016) report, twenty-two percent of women of reproductive age were undernourished, leaving their children predisposed to low birth weight, short stature, low resistance to infections, and high risk of disease and death. Of the total childbearing-age female deaths, 25 percent were related to pregnancy or childbearing. The prevalence of stunting and acute malnutrition (wasting or low weight-for-height)in Ethiopia has decreased over the past decade but remains high, with 38 percent(5.8 million) of children under 5 years stunted and 10 percent waste(Central Statistical Agency (CSA) ., 2016)*.* But the prevalence of anemia in childbearing-age females was increasing from 2011 (17% to 24%) to 2016.

Therefore, the government of Ethiopia recognized nutritional problems as a major public health issue and made global and regional commitments to address nutritional problems (FDRE, 2016). For example, the July 2003 Maputo Declaration of Comprehensive Africa's agriculture development program, the Scaling Up Nutrition (SUN) Movement 2013, the global Nutrition for Growth compact made at the 2013 Nutrition for Growth summit in London, and the African Union’s Malabo Declaration on accelerated agricultural growth and transformation for shared prosperity and improved livelihoods can be mentioned.

Similarly, the Ethiopian government also made national commitments and strategies like the five-year national nutrition strategy (2008), growth and transformation plan II (GTP), the revised second national nutrition program (NNP II) (2016–2020), and the Seqota Declaration (2015). In particular, the Seqota Declaration aims to end hunger (zero stunting) and undernutrition by 2030 through nutrition-sensitive and nutrition-specific programs. Despite these efforts, maternal and child malnutrition is still unacceptably high(FDRE, 2008, FDRE, 2016b, USAID, 2018).

In Ethiopia, even though many epidemiological studies reported on the nutritional status and dietary practices among pregnant women(Daba, Beyene et al. 2013, Kedir, Berhane et al. 2013, Moges, Worku et al. 2015, Zelalem, Endeshaw et al. 2017, Workneh, Eglovitch et al. 2021), there are very scanty nutritional interventions reported (Diddana, Kelkay et al. 2018). To our knowledge, there is no documented study carried out to evaluate the effects of nutritional education and home gardening interventions among pregnant women on feto-maternal outcomes in the study area. Thus, this study aimed to assess the synergistic effects of community-level nutrition education and home gardening interventions on feto-maternal health outcomes among pregnant women in Jimma Zone, Southwest Ethiopia. It would further help in designing policies and programs that target pregnant women to improve their fetal and maternal health outcomes. Furthermore, it would be helpful in the planning and implementation of nutritional interventions. It would also add to the global literature on the integration effect of nutrition-sensitive and specific approaches during pregnancy on fetal and maternal health outcomes.

The following conceptual framework was adapted from different works of literature to show the relationship between various variables considered in the study


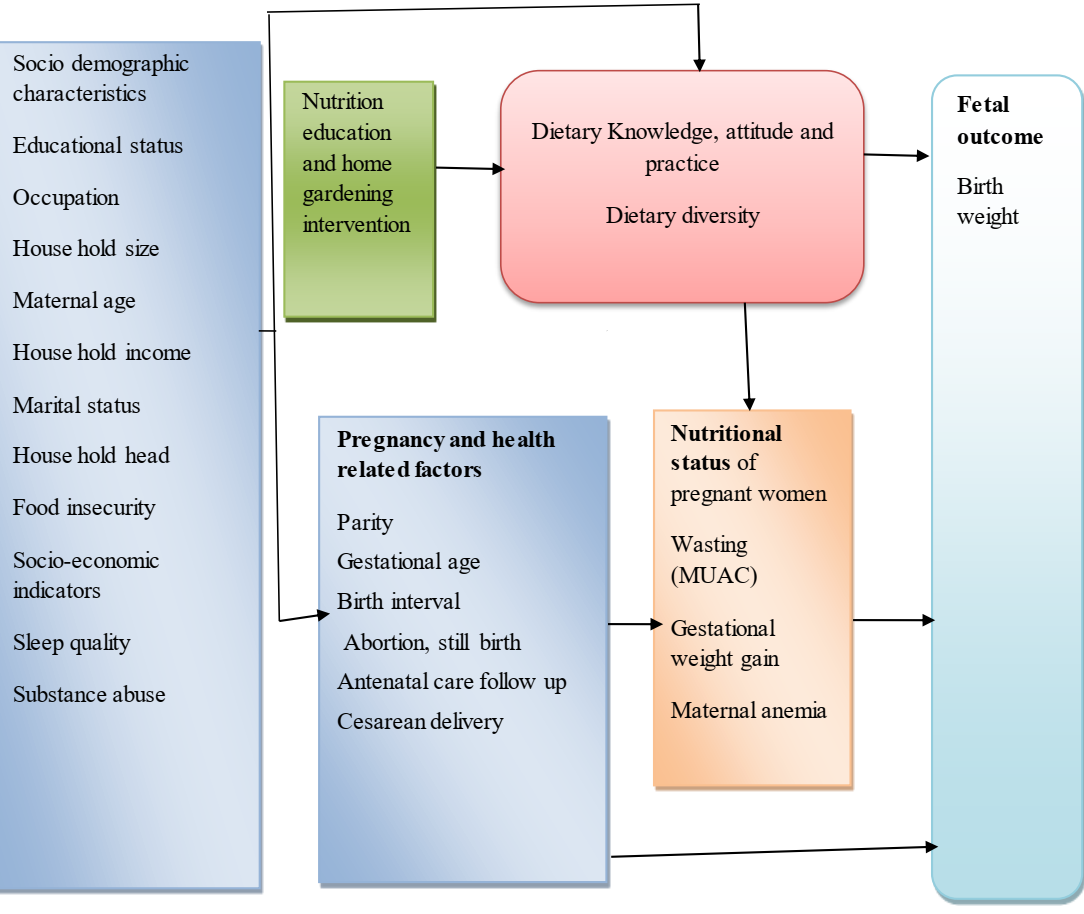


[Figure 2. Map of the study area with selected study clusters (kebeles) of both districts (Seka and Mana) source: Generated using ArcGIS software version 10.3 from ETHIO-GIS, 2014 database. 21](#_Toc137962176)

[Figure 3. Shows that the sampling procedure for the effect of home gardening and social behavioral change communication on feto-maternal outcomes in Jimma zone, South West Ethiopia, 2019. 22](#_Toc137962177)

,*Olney et al 2003*, *Herforth A, 2016,Rule M T, et al , 2018)*.

## Research hypothesis

1. Pregnant women who are involved in nutrition education and home gardening interventions will have a better dietary diversity score and nutritional status than the controls.
2. Pregnant women who are involved in nutrition education and home gardening interventions will give more normal birthweight neonates than the controls.

# CHAPTER Two: OBJECTIVES

## 2.1. General objective

This study aims to assess the effect of nutritional education and home gardening interventions on feto-maternal outcomes among pregnant women in Jimma Zone, Southwest Ethiopia.

## 2.2. Specific objective

- 1. To measure the effect of nutritional education and home gardening interventions on minimum dietary diversity scores among pregnant women of Jimma zone.
  2. To determine the effect of nutritional education and home gardening interventions on the nutritional status among pregnant women of Jimma Zone.
  3. To determine the effect of nutritional education and home gardening interventions during pregnancy on fetal birth weight in Jimma zone.

# CHAPTER THREE: METHODS AND MATERIALS

## 3.1. The study settings

A cluster randomized controlled trial will be conducted in two selected districts of Jimma zone, representing the two known agroecological (coffee growing and food crop growing) areas of the zone, based on the type of mainly produced crop in the area. Accordingly, Mana is selected from coffee-growing districts. Khat is also another important cash crop in this area. Mana is found at on1911 meters of altitude above sea level with an estimated total population of 160,096, of whom 80,481 are men and 79,615 are women. Mana district has 26 kebeles (small administrative units) including Yebu and Bilida towns). The other selected district is Seka Chekorsa from a grain and food crop-producing area (like; fruits, teff, peppers, and Khat). It has an altitude between 1580 to 2560 meters above sea level and an estimated total population of 336,277, of whom 168,863 were males and 167,414 were females. This district has 37 kebeles including the two town kebeles of Seka. Generally, Jimma zone is considered one of the leading coffee-producing zones of the Oromia regional state. The annual rainfall of the zone range between 1200-2800 mm per annum. In normal years, the rainy season extends from February to November. Also, it has eye-catching historical places like the Palace of King Aba Jifar, the Jiren Mountain, and Seka Falls. The zone is found in southwest Ethiopia ∼345 km from the capital city of the country Addis Ababa. The study will be conducted from February 2019 –November 2019.

## 3.2. Source population

All first-trimester pregnant mothers of Jimma Zone within the age group of 15 – 49 years old during the study period will be the source population.

### 3.2.1*.* Study population

All first-trimester pregnant mothers of the selected clusters (kebeles) during the study period will be the study population.

### 3.2.2. Study Units

The selected study clusters will be the study unit. First-trimester pregnant mothers who will participate in the study will be a unit of analysis. Mothers who will participate in the study will be identified by doing home to a home census of the study clusters and fulfilling the criteria.

### 3.2.3. Inclusion criteria

All first-trimester pregnant mothers in the selected study area (cluster) who planned to give birth in the zone and are willing to give consent will be included.

### 3.2.4. Exclusion criteria

First-trimester pregnant mothers of the selected study clusters who have known (self-reported) chronic medical diseases (like diabetes mellitus, CHF, renal failure, epilepsy, HIV/AIDS) and temporary residents who will not stay in the study area until delivery. Mothers who are not willing to give consent for participation in the study and have multiple pregnancies after ultrasound scanning will be also excluded.

## 3.3. Study design

The study design will be a cluster-randomized controlled trial involving two arms. There will be intervention and control groups. The study clusters will be identified and arranged on the map first, by considering a buffer zone in between avoid information contamination. After all, the selected clusters will be assigned randomly either to the intervention or control groups ([See the map).](#_4.5._Sampling_procedures)

## 3.4. Sample Size Determinations

The sample size was estimated by G power version 3.1.9.7. Assumptions used to calculate the required sample size were: precision of 5%, power of 80%, an effect size of 0.25, and we expect a mean birth weight change of 100 gm (the primary outcome) in the intervention groups (from 2975 g to 3075 g) based on previous studies. Design effects of 2 and 15% non-response rates were also considered. The total sample size was 348 pregnant women (116 per each of the three study arms).

The sample size for each study cluster (kebele) will be allocated based on the proportion of their respective population. Accordingly, a total of eighteen clusters (kebeles) will be selected. From Mana and Seka districts eight and ten clusters (kebeles) respectively.

## 3.5. Sampling procedures

Two districts (woreda) that could represent the two known agroecological areas of the Jimma zone will be selected. Accordingly, Mana from predominantly coffee-growing districts and Seka Chekorsa from grain and crop-growing districts were selected purposively for management and logistical reasons. Then, non-adjacent clusters or kebeles (the smallest administrative units) will be selected from both districts to have buffer zones**.** Accordingly, a total of 18 clusters (8 from Mana and 10 from Seka chekorsa) districts will be selected. Using the randomized complete block design; the clusters (kebeles) will be grouped into two blocks based on their agroecological areas (mainly coffee or grain producing). Finally, from both blocks clusters will be randomly assigned to the three study arms (husband, Peers, and control). Thus, Nase, Bidaru toli, Dimtu (of Seka), Doyo toil, Gudata bula, and Lemi Lelisa clusters(kebeles) will be assigned to the husband group. While, Siba bake, Buyo kechema, Meti, Bore, Bebela kosa, and Haro clusters will be assigned to the peer group. Likewise, Komo hare, Gepa seden, Wakito madalu, Kamise waraba, kore Lelisa, and kenteri clusters will be assigned to the control group.

Regarding the sample size allocation, it was proportionally to the population size of respective districts and kebeles (clusters). A total of 348 first-trimester pregnant women will be enrolled at baseline, 144 women will be from the mana district (48 women in each arm), and 204 women will be from the Seka-chekorsa district (68 women in each arm). This means that a total of 116 1^st^ trimester pregnant women per the three study arms will be enrolled. All of the first-trimester pregnant women who will fulfill the inclusion criteria in each cluster will be enrolled until the desired or allocated number will be achieved.


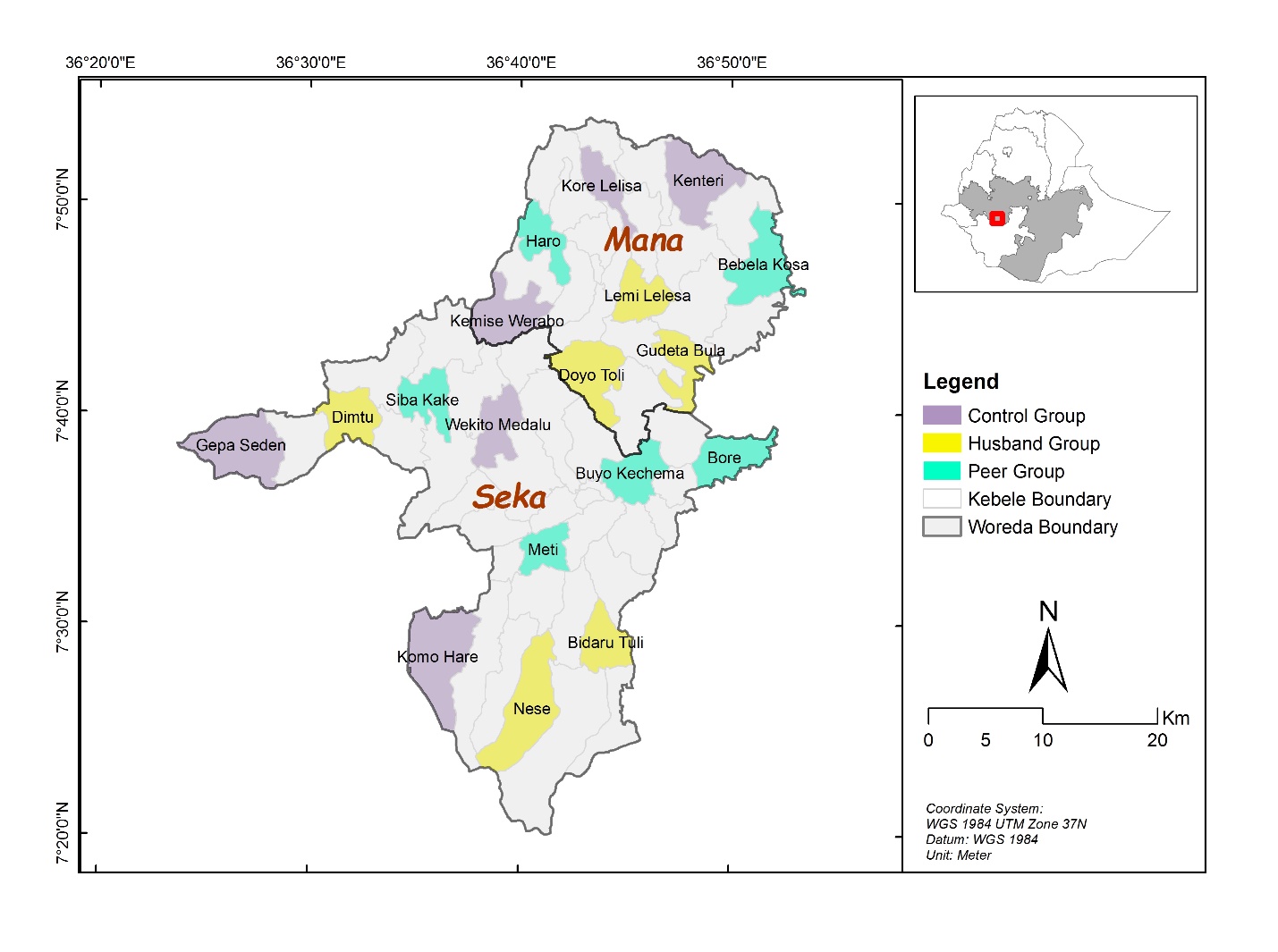


Figure 2. Map of the study area with selected study clusters (kebeles) of both districts (Seka and Mana) source: Generated using ArcGIS software version 10.3 from ETHIO-GIS, 2014 database.

Jimma zone

21 Districts ( woredas)

Goma,

Limu kosa

Mana

Gera

Gumay

Limu seka

Boter –tolay

Chora- boter

Shebe- sombo

Non benja

Omo- Nada, Omo-Beyam

Sekachekorsa

Mancho

Dedo

Kersa

Tiroafata

Sokoru,

Santama

Sigimo

Gatira

Predominantly Grain/crops producing districts.

11 districts

---

Predominantly coffee growing districts,

10 districts

Mana district selected

Mana district

26 kebeles (clusters) total

Sekachekorsa district

37 kebeles (clusters) total

10 Clusters selected

8 Clusters selected

Coupled Intervention arm

3 clusters

Coupled intervention arm 3clusters

Mothers with their peers arm

4 clusters

Control arm

3clusters

Mothers with their peers arm

2 clusters

Control arm

3 clusters

Figure 3. Shows that the sampling procedure for the effect of home gardening and social behavioral change communication on feto-maternal outcomes in Jimma zone, South West Ethiopia, 2019.

Total=18 clusters

1. Husband intervention arm=6 clusters/kebeles (Nese, Gepa seden, Dimtu, Doyo toil, Gudata bula, Lemi lelise
2. Mothers with their peer intervention arm=6 clusters/kebeles (Siba beke, Meti,Buyo kechema, Bore,Babala kosa, Haro
3. Control arm = 6 clusters/kebeles (Bidaru tuli,Komo hare,Wakito medalu,Kemise waraba,Kore lelisa,Kenteri

## 3.6. Study Variables

### 3.6.1. Dependent variables

- Birth weight
- Minimum dietary diversity scores
- Wasting (MUAC) and anemia
- Gestational Weight gain
- Nutritional Knowledge, attitude, and practice

### 3.6.2. Independent variables

- Socio-Demographic characteristics: Maternal age, educational level, occupation, household size, household income, and status of women in the households.
- Obstetric factors like; inter-birth interval, gestational age, parity, gravidity, abortion, and other pregnancy-related complications.

## 3.7. Timing and outputs of fieldwork

### 3.7.1. Preparatory phase

The duration of the intervention will be nine months. It will be started from Augest 2020 through December 2021. In this phase, training of counselors (midwife/Nurse) from each nearby health center/hospital for each cluster will be done. The training will equip nutrition counselors with skills and knowledge of counseling on the below-selected topics of sessions. In the same way, one community development agent per cluster will be trained to support pregnant mothers. The training of the development agent will cover topics about home garden vegetables land preparations, watering; weed control, cultivating, pest control, and disease control. It will be given for seven days by agricultural experts. In addition to the above activities, a community-based survey of first-trimester pregnant women (missed two periods) in the selected clusters/ kebeles/ will be done by health extension workers is also part of this phase.

### 3.7.2. Intervention phase

This is designed to answer the objective of the dissertation. It includes two components.

**The Social and behavioral change communication component**

This component aims to equip pregnant women and their husbands with action-oriented knowledge, behavior, and attitude that will motivate their ability to adopt optimal dietary diversity and healthcare practices for healthy feto-maternal outcomes. Different approaches to delivering education, information, and counseling techniques will be used during the secessions. Accordingly, lecture methods (direct information provision by the counselor), question and answering of unclear ideas, sharing experiences, discussions, demonstrations, telling stories, as necessary using posters, and provision of prepared leaflets will be used. At the end of each session, a summary of dietary diversity's importance to increasing the intake of nutrient-dense diets, clean drinking water, and personal and environmental hygiene importance for healthy maternal and neonatal outcomes will be a key message. Behavioral change communication (SBC) will be given three times by trained counselors. The time of delivery will be; at the begging of the intervention (during enrolment), during the second and third-trimester normal antenatal care follow-up appointments. The duration per session of education will be 1-2 hours***.***

Topics for Session 1

1. Minor disorders of pregnancy and their management.
2. Importance of dietary diversity
3. Importance of weight gain during pregnancy
4. Importance of personal and environmental hygiene
5. Importance of IFA supplementation.
6. Use of bed nets and TT vaccination
7. Importance for daytime rest during pregnancy.
8. Additional meal importance during pregnancy.
9. Importance of Iodized salt use during pregnancy.

Topics for Session 2

1. Reinforcement of the previous topics.
2. Birth preparedness and complications redness. (needs preparation for birth, place of delivery, skilled attendants for delivery, finance, transport, blood donation, emergency plan)
3. Danger signs of pregnancy that need immediate medical care.

Topics for Session 3

1. Reinforcement of previous topics.
2. Signs of Labor and delivery
3. Early and exclusive breastfeeding and colostrum
4. Institutional delivery advantages
5. Complications of the postpartum period for both (mother and neonate)

**The Home Gardening Component**

Regarding the home gardening part, the intervention groups will be provided or supplemented with four vegetable seeds (lettuce, tomato, cabbage, and carrot) which were selected by professionals consultation about the nutritious availability, and suitability to the agro-ecology of the zone, to reach for consumption (mature) within three months**.** The vegetable seeds (home garden seeds), will be supplied by identified private sector seed suppliers or farmers’ cooperatives in the respective woredas. The mother and her husband should have clearly understood that this is not mean that the pregnant mother should have to eat only in the home gardens prescribed here. But, this is just to increase the availability of various food sources in addition to what they have. Therefore, pregnant mothers should have got a variety of foods from both animals and plant sources will be given as the core message. The community development agent of the cluster will guide them from land preparation to harvest through weekly home visits. The home gardening activity should have to be done by the husband or either family members other than the pregnant mother will be remembered to avoid acquiring toxoplasma Gondi.

### 3.7.3. Monitoring and Evaluation Phase

Monitoring of intervention implementation will be carried out through supervisory visits to the study area by the principal investigator and monthly meetings with the team. It will be enhanced by recording, monitoring activities, and process evaluations. This is to confirm the progress of the interventional implementation process; whether the planned activities were implemented as scheduled, and how the pregnant mother and her family (husband) accepted and carry out the interventional activities. Also, it is for the provision of a necessary correction on time if contextual barriers will be identified during the intervention. The corrective adjustments taken during the visit will be documented. Evaluation of overall activities will be done in the middle (during late 2^nd^TMP to 3^rd^TMP) and at the last. ON

### 3.7.4. Compliance

The number of mothers who attended the health education sessions and the no. of mothers who harvest the home gardens as ordered will be followed. Also, scheduled home visits will be made by the research team to confirm the level of performance by identifying the number of mothers who comfortably attended the health education sessions and starts to carry out, the number of mothers on implementations of home gardens cultivation as guidance by the development agent. Both the intervention and control groups will receive the routine nutritional education provided by antenatal care follow-up providers as usual.

Table 2: Shows the summary of main interventional activities protocol of the effect of home gardening and social behavioral change communications on fetal outcomes in Jimma Zone, Southwest Ethiopia, 2019.

| Key action(message) | | Strategy of intervention | Responsible person | Frequency | Compliance Parameter |
| --- | --- | --- | --- | --- | --- |
| Nutritional education | Session one | Direct information provision  Sharing experiences  Discussions  Demonstrations  Telling stories  leaflets with the key message (Afan Oromo) | Trained counselors | Once during enrolment for  1-2hrs | A number of women and husbands attended nutrition education.  A number of women received the leaflet. |
|  |  |  |  |  |  |
|  | Session two | Same as above | Trained counselors | Once during the second trimester for  1-2 hours. | A number of women and husbands attended nutrition education. |
|  | Session three | Same as above | Trained counselors | Once during the third trimester for  1-2hrs hours | A number of women and husbands attended the education. |
|  | Home visiting | Provide information  Encourage  Correct | Trained counselors, peers, and PI | Monthly | The number of women visited |
| Home Gardening | Seeds provision  (lettuce, tomato, cabbage, and carrot) | Provided by preparing one teaspoon of each seed/woman. | PI and DA | Once | The number of mothers who received seeds |
|  | Land preparation and care for home gardens | Demonstrations | DA | Once | A number of women and their husbands attended the demonstrations |
|  | Home visiting | Encourage  Show  Advice  Correct | DA | Fortnight | The number of women prepared the land, planted the seeds, and used the vegetables |

*Footnote: PI; principal investigator, DA; Agricultural development agent*

## 3.8. Data collection and instruments

The data will be collected in two phases. After the home-to-the-home census of eligible pregnant mothers enrolment, the baseline data will be collected by using pre-tested structured questioners, and guidance equations. Accordingly, data on socio-demographic characteristics, obstetrics history, MUAC, Hgb level, and 24-hour recall minimum women dietary diversity scores (FAO and FHI guideline 2016) will be collected. To get the best possible information, first, the mother will be asked to narrate what she remembers followed by probes to ascertain that no meal or snack will be left out. After all, a detailed list of all the ingredients of the dishes, snacks, or other foods consumed will be generated and grouped into ten. The end-line data will be collected after the mother gave birth, just within 24 hrs.

## 3.9. Data Quality Control

The baseline and end-line data will be collected by experienced and trained eight female midwives/nurses/ who are well familiar with the study's geographical area and can speak the local language (Afan Oromo. They will receive seven days of training on the data collection instruments, how to record the response of the mother, and anthropometric measurements. The training will be conducted just before the onset of the study followed by practical tests to make sure that skills were appropriately transferred. Also, a standardization exercise will be performed during the training to capture the technical errors in measurements. In each selected cluster, one trained supervisor will be assigned to give on-site support and oversee the completeness of collected data overnight. Also, the principal investigator will be made a weekly visit to check the completeness and quality of the collected data.

## 3.10. Pretest

All teams of the data collection will be involved in the pretest assessment. It will be conducted on 90 pregnant mothers two weeks before the real data collection in kersa district. This district is almost similar in socio-demographic and agroecological to the selected study area. This will enable us to check the reaction of respondents to the procedure and to test the data collection instrument for clarity, time consumption, and weather appropriately collect the intended information. Also, the appropriateness of statistical analysis ways will be tested. Finally, adjustments will be made based on the findings as necessary

## 3.11. Dietary intake assessment

To determine a minimum dietary diversity score, a single point will be awarded to each food group consumed over the reference period, and a total of all points calculated. The dietary diversity will be calculated by using the minimum DD-women (MDD-W) indicator, which is an improved version of the Women's Dietary Diversity (WDD) score and has 10 food groups, consumption of at least 5 of which indicates high dietary diversity. In addition, a multiple-pass 24 hrs recall method will be used to improve memory.

## 3.12. Anthropometric measurements

The pregnant mothers will be weighed at the begging and last ANC follow-up without shoes and with minimal or light clothing using a Seca digital scale. The readings will be taken to the nearest 0.1kg. It will be calibrated against known weight regularly. MUAC will be measured during the enrolment and at the end of the follow-up to assess wasting among the study subjects using an adult MUAC non-stretchable measuring tape to the nearest 0.1 cm. The Mid-upper arm circumference will be measured midway between the lateral projection of the acromion process of the scapula and the inferior margin of the olecranon process of the ulna.

## 3.13. Hemoglobin level Determination

Hemoglobin will be measured at baseline and end line using a portable, battery-operated photometer (HemoCue). After swiping the site with disinfectant, a finger prick will be made to obtain blood for hemoglobin measurement. The first two drops will be swapped away and the third drop will be used to fill the micro cuvette for reading the hemoglobin. The machine will be calibrated daily with a reference microcuvette provided with the machine. A trained and skilled laboratory technologist will be responsible for the collection of blood samples and hemoglobin determination.

## 3.14. Statistical analysis

Data will be entered into Epi-data Version 3.1 for cleaning and sorting and then exported to SPSS version 20 for analysis. First, we will present descriptive information on the socio-demography of enrolled mothers, and then, baseline measurements will be compared between allocation groups by using mean difference or chi-squares. Comparison of the KAP scores, birth weight, hemoglobin level, and GWG, differences among the study arms will be done using ANOVA. The normality assumptions and the homogeneity of variance will be checked using a Q-Q plot and Levene’s test. The linear GEE regression analyses will be used to test the effects of the interventions on feto-maternal outcomes (birth weight and hemoglobin level) of difference in difference.

## 3.15. **Ethical Considerations**.

The study protocol will be approved by the institutional review boards of Jimma University Institute of health science and the Oromia Regional Health Bureau. The zonal health office, the woreda administrators, and the local authorities will be informed about the research through an official letter from the University to obtain their permission. Verbal informed consent will be obtained from the eligible participants after a detailed explanation of the purpose and methods of the study. Also, they will be informed that they are free to withdraw from the study at any time when they are not comfortable with it. Confidentiality of the data will be secured by not using any personally identifiable information on the questionnaires and by locking the questionnaires in a secured cabinet. Personal identifiers and the other parts of the data will be kept in separate areas.

# CHAPTER Four: Budget

**Personnel costs**

For trainer=7 days*1000*2 personnel for (health extensions) =14000

For trainees=18(clusters*7 days) HEW*300=37800

Trainer= 7 days*1000/day*2personel for community development agent= 14000

Trainees=18 clusters*7 days for DA*300=37800

Home visit weekly by =100/day*for 38 weeks*18 clusters*2 persons=136800

For monthly supervision=9 moths*1000/day*2 individuals=18000

Total=258,400

**Data collection**

Data collectors’ trainer=2 *7 days*1000birr/day=14000

Data collectors’ supervisors=6*14 days*300/day=25200

Data collectors training=7 days*8 individuals*300 birr/day=16800

Data collection=for 2weeks*2(times)*8 individuals *300=67200

Total=123,200

**Transportation costs**

Fuel=10,000

During the training of peers and DA=48 individuals*100 birr*2=9600

For data collectors and supervisors=8*100+6*100= 2800*2times= 5600

Total=25200

**Supplies costs**

Ultrasound rent=1000 birr/day for 30 days=30000 birr

Seeds = varieties of seeds*400 mothers*200 birr= 80000

Teaching materials=leaflets 400*2birr=800birr

Photocopy=1000 birr

Paper=300*6=1800 birr

Pen=5pac*300 birr=1500

Pencil=6pack*200 birr=1200

Laboratory reagents and materials=50,200

Total=166500

## 4.1. Budget Summary

Table 4. Shows the budget summary of the effect of Home gardening and Social Behavioral change communication on feto-maternal outcomes in Jimma Zone, South West Ethiopia, 2019.

| S/N | Budget Category | Total Cost |
| --- | --- | --- |
| 1 | Personnel | 258400 |
| 2 | Transport | 25200 |
| 3 | Supplies | 116300 |
| 4 | Lab. Test | 50,200 |
| 5 | Data collection | 123200 |
| Total |  | 548300 |
|  | Contingency (10 %) | 54830 |
| Grand Total Cost | | 603130 |

# CHAPTER Five: Work plan

Table 5.Shows the work plan of the effect of Home gardening and Social Behavioral Change communication on feto-maternal outcomes in Jimma Zone, South West Ethiopia, 2019.

| Ser. | Activities | Responsible person | 2019 | | 2020 | | | | 2021 | | |
| --- | --- | --- | --- | --- | --- | --- | --- | --- | --- | --- | --- |
|  |  |  | Jul-Sep | Oct-Dec | Jan -Mar | Apr-Jun | Jul-Sep | Oct-Dec | Jan-Mar | Apr-Jun | Jul-Sept |
| 1 | Proposal preparation | PI |  |  |  |  |  |  |  |  |  |
| 2 | Proposal submission, feedback | PI and CIs |  |  |  |  |  |  |  |  |  |
| 3 | Ethical approval | JU |  |  |  |  |  |  |  |  |  |
| 4 | Budget secured | PI, CIs |  |  |  |  |  |  |  |  |  |
| 5 | Data collectors training | PI |  |  |  |  |  |  |  |  |  |
| 6 | Mapping of the study site | PI |  |  |  |  |  |  |  |  |  |
| 7 | Baseline data collection | PI, DCs |  |  |  |  |  |  |  |  |  |
| 8 | Nutrition education and seeds distributions | DCs |  |  |  |  |  |  |  |  |  |
| 9 | Baseline data entry and analysis | PI |  |  |  |  |  |  |  |  |  |
| 10 | Preliminary data publication | PI, CIs |  |  |  |  |  |  |  |  |  |
| 11 | Feto-maternal outcomes  data collection |  |  |  |  |  |  |  |  |  |  |
| 12 | Feto-maternal outcomes  data analysis | PI |  |  |  |  |  |  |  |  |  |
| 13 | Second and third publications | PI, CIs |  |  |  |  |  |  |  |  |  |
| 15 | Analysis of overall study | PI |  |  |  |  |  |  |  |  |  |
| 16 | Draft submission | PI |  |  |  |  |  |  |  |  |  |
| 17 | Final presentations | PI |  |  |  |  |  |  |  |  |  |
| 18 | Dissemination of finding and final paper publication | PI |  |  |  |  |  |  |  |  |  |

# CHAPTER Six: Reference

A., M. (2018) ‘RESEARCH ARTICLE PREVALENCE OF LOW BIRTH WEIGHT IN HOWRAH .’, *. International Journal of Current Research*, 10(6), pp. 69993–69998.

A. Talukder, N.J. Haselow, A.K. Osei, E. Villate, D. Reario, H. Kroeun, L. SokHoing, A. Uddin, S. D. and V. Q. (2010) *Homestead food production model contributes to improved household food security and nutrition status of young children and women in poor populations.*, *Urban Agriculture*.

Al-Jemil N, Tabassum H, Ali MN, Abudul Qadeer M, A. K. F. and A. R. M. (2017) ‘Correlation between serum trace elements and risk of preeclampsia :A case controlled study in Riyadh, Saudi Arabia .’, *Saudi Journal of Biological Sciences.*, 24(6), pp. 1142–1148. doi: https://doi.org/10.1016/j.sjbs.2015.02.009.

Ali F, Thaver I, K. S. (2014) ‘Assessment of dietary diversity and nutritional status of pregnant women in ISLAMABAD , PAKISTAN .’, *J Ayub Med Coll Abbottabad*, 26(4), pp. 506–9.

Amiri P, Z. N. H. and N. P. et al. (2017) ‘Can an Educational Intervention Improve Iodine Nutrition Status in Pregnant Women. A Randomized Controlled Trial .’, *Thyroid*, 27(3). doi: 10.1089/thy.2016.0185.

Asayehu, Tamene Taye, C. L., Henauw, S. De and Gebreyesus, S. H. (2017) ‘Dietary behaviour, food and nutrient intake of women do not change during pregnancy in Southern Ethiopia’, *Maternal &amp; Child Nutrition - Wiley Online Library*. John Wiley & Sons, Inc, 13(2). doi: https://doi.org/10.1111/mcn.12343.

Asefa, F. and Nemomsa, D. (2016) ‘Gestational weight gain and its associated factors in Harari Regional State .’, *Reproductive Health*. Reproductive Health, 13(101), pp. 1–7. doi: 10.1186/s12978-016-0225-x.

Asi L N, Teri DT,Meyer-rochow, V. B. (2018) ‘Influence of food taboos on nutritional patterns in rural communities in Cameroon’, *International Review of Social Research*, 8(1), pp. 2–6. doi: https://doi.org/10.2478/irsr-2018-0013.

Assefa N, Berhane Y, and W. A. . (2012) ‘Wealth Status, Mid Upper Arm Circumference (MUAC) and Antenatal Care (ANC) Are Determinants for Low Birth Weight in Kersa, Ethiopia.’, *PLoS ONE*, 7(6), p. e39957. doi: doi.org/10.1371/journal.pone.0039957.

Barker D J.P, Gelaw J, and Th. et al. (2010) ‘The early origins of chronic heart failure: Impaired placental growth and initiation of insulin resistance in childhood’, *European Journal of Heart Failure*, pp. 819–825. doi: 10.1093/eurjhf/hfq069.

Berti, P. R., Krasevec, J. and FitzGerald, S. (2004) ‘A review of the effectiveness of agriculture interventions in improving nutrition outcomes’, *Public Health Nutrition*, 7(5), pp. 599–609. doi: 10.1063/1.4736925.

Bhutta A Z, asMBA J, Rizvi A, Gaffey F M, WALker N et al and the Lancet Nutrition Interventions Review Group, the M. and C. N. S. (2013) ‘Evidence-based interventions for improvement of maternal and child nutrition. what can be done and at what cost?’, *The Lancet*, 452–477(9890), p. 382. doi: https://doi.org/10.1016/S0140-6736(13)60996-4.

Black, R. E. *et al.* (2013) ‘Maternal and child undernutrition and overweight in low-income and middle-income countries’, *The Lancet*, pp. 427–451. doi: 10.1016/S0140-6736(13)60937-X.

Black M M, Pérez-Escamilla R, and R. S. F. (2015) ‘Integrating Nutrition and Child Development Interventions. Scientific Basis, Evidence of Impact, and Implementation Considerations.’, *Adv Nutr.*, 6(6), p. 852–859. doi: 10.3945/an.115.010348.

Black M M, Walker S P, F. C. P. H. et al and for the L. E. C. D. S. S. C. (2017) ‘Advancing Early Childhood Development: from Science to Scale 1.’, *Lancet.*, 389(10064), p. 77–90. doi: 10.1016/S0140-6736(16)31389-7.

Central Statistical Agency (CSA) [Ethiopia] and ICF. (2016) *Ethiopia Demographic health survey 2016.* Addis Ababa, Ethiopia, and Rockville, Maryland, USA.: CSA andICF.

Darnton-Hill, I. (2012) ‘Global burden and significance of multiple micronutrient deficiencies in pregnancy’, *Nestlé Nutrition Institute workshop series*, pp. 49–60. doi: 10.1159/000337421.

Demissie T, and K.-M. W. (1998) ‘Food taboos among pregnant women in Hadiya Zone.’, *The Ethiopian Journal of Health Development*, 12(1), pp. 45–49.

Development intiatives. (2017) *The Global Nutrition Report 2017.*, *Development Initiatives*. Bristol, UK.

Diana R , Rachmayanti R D, Anwar F, Khomsan A, and C. D. F. (2018) ‘Food taboos and suggestions among Madurese pregnant women : a qualitative study’, *Journal of Ethnic Foods*. Elsevier Ltd, pp. 1–7. doi: 10.1016/j.jef.2018.10.006.

Diddana, T Z, Kelka G N ,Dola A N, and S. A. (2018) ‘Effect of Nutrition Education Based on Health Belief Model on Nutritional Knowledge and Dietary Practice of Pregnant Women in Dessie Town, Northeast Ethiopia: A Cluster Randomized Control Trial.’, *Journal of Nutrition and Metabolism*, p. 10. doi: 10.1155/2018/6731815.

E, W. and Maria, S.-R. A. (2010) ‘Prenatal nutrition: A practical guide for assessment and counseling’, *Journal of Midwifery and Women’s Health*, pp. 540–549. doi: 10.1016/j.jmwh.2010.06.017.

Endeshaw, M. *et al.* (2014) ‘Effect of Maternal Nutrition and Dietary Habits on Preeclampsia : A Case-Control Study’, *International Journal of Clinical Medicine.*, (5), pp. 1405–1416. doi: 10.4236/ijcm.2014.521179.

Fallah F, Pourabbas A, Delpisheh A, V. Y. and S. M. (2013) ‘Effects of Nutrition Education on Levels of Nutritional Awareness of Pregnant Women in Western Iran.’, *Int J Endocrinol Metab.*, 11(3), pp. 175–178.

FDRE (2016a) *FEDERAL DEMOCRATIC REPUBLIC OF ETHIOPIA NATIONAL NUTRITION*.

FDRE (2016b) ‘SEQOTA DCLARATION:Implementation Plan ( 2016 – 2030 )’. Addis Ababa: Federal democratic repubilic of ethiopia, p. 91.

Federal ministry of health of ethiopia (2008) ‘Federal Democratic Republic of Ethiopi National Nutrition Strategy’. Addis Ababa, pp. 1–17.

Gebremedhin S, Baye K, BekeleT, Tharaney M, Asrat Y, A. Y. and R. N. (2017) ‘Predictors of dietary diversity in children ages 6 to 23 monthes.’, *Elsevier*, 33, pp. 163–168. doi: https://doi.org/10.1016/j.nut.2016.06.002.

Gelli A, Becquey E, Ganaba R, Headey D, Hidrobo M, et al . . (2017) ‘Improving diets and nutrition through an integrated poultry value chain and nutrition intervention (SELEVER) in Burkina Faso study protocol for a randomized trial.’, *Trials*, 18, p. 412. doi: 10.1186/s13063-017-2156-4.

George M. Weisz and William R. Albury (2014) ‘Hunger Whilst “In Utero” Programming Adult Osteoporosis’, *Rambam Maimonides Med J*, 5(1), p. e0004. doi: [10.5041/RMMJ.10138.

Getnet W, Aycheh W, and T. T. (2018) ‘Determinants of Food Taboos in the Pregnant Women of the Awabel District, East Gojjam Zone, Amhara Regional State in Ethiopia.’, *Advances in Public Health*, 2018, p. 6. doi: 10.1155/2018/9198076.

Gicevic S, G. A. J. and F. T. et al (2018) ‘Evaluating pre-pregnancy dietary diversity vs. dietary quality scores as predictors of gestational diabetes and hypertensive disorders of pregnancy.’, *PLoS One*, 13(4), p. e0195103. doi: [10.1371/journal.pone.0195103].

Girard A W, Self J L, McAuliffe C, and O. O. (2012) ‘The Effects of Household Food Production Strategies on the Health and Nutrition Outcomes of Women and Young Children : A Systematic Review’, *Paediatric and perinatal Epidemiology.*, 26(1), pp. 205–222. doi: 10.1111/j.1365-3016.2012.01282.x.

Gizaw B, and G. S. (2018) ‘Factors associated with low birthweight in North Shewa zone, Central Ethiopia’, *Italian Journal of Pediatrics*.

Goshu, H., Teshome, M. S. and Abate, K. H. (2018) ‘Maternal dietary and nutritional characteristics as predictor of newborn birth weight in Jimma Town , Southwest Ethiopia .’, *Journal of Public Health and Epidemiology*, 10(May), pp. 155–164. doi: 10.5897/JPHE2017.0977.

Grum T, Seifu A, Abay M, A. T. and T. L. (2017) ‘Determinants of pre-eclampsia_Eclampsia among women attending delivery Services in Selected Public Hospitals of Addis Ababa, Ethiopia.’, *BMC Pregnancy and Childbirth*. doi: https://doi.org/10.1186/s12884-017-1507-1.

Hadush Z,Birhanu Z,Chaka M, and G. H. (2017) ‘Foods tabooed for pregnant women in Abala district of Afar region , Ethiopia : an inductive qualitative study’, *BMC Nutrition*. BMC Nutrition, pp. 1–9. doi: 10.1186/s40795-017-0159-x.

Haji K, Yemane B, and A. W. (2013) ‘Khat Chewing and Restrictive Dietary Behaviors Are Associated with Anemia among Pregnant Women in High Prevalence Rural Communities in Eastern Ethiopia.’, *PLoS ONE*, 8(11), p. e78601.

Hawkes C, and F. J. (2017) *Nourishing the SDGs: Global Nutrition Report 2017*. Bristol: Development Initiatives Poverty Research Ltd. Available at: http://openaccess.city.ac.uk/id/eprint/19322.

Herforth A, and B. T. J. (2016) ‘Nutrition indicators in agriculture projects : Current measurement , priorities , and gaps’, *Global Food Security*. Elsevier, 10, pp. 1–10. doi: 10.1016/j.gfs.2016.07.004.

Hu, F. B. (2002) ‘Dietary pattern analysis_ a new direction in nutritional epidemiology.’, *Current Opinion in Lipidology.*, 13(1), pp. 3–9.

Hutagalung, L. (2017) ‘ANEMIA AND NUTRITIONAL STATUS AS DOMINANT FACTOR OF THE EVENT LOW BIRTH WEIGHT IN INDONESIA : A SYSTEMATIC REVIEW’, *LIFE: International Journal of Health and Life-Sciences*, 3(1), pp. 29–38. doi: DOI-https://dx.doi.org/10.20319/lijhls.2017.31.2938 ANEMIA.

Janet C. King (2003) ‘Risk of Maternal Nutritional Depletion and Poor Outcomes Increases in Early or Closely Spaced Pregnancies _ _ Oxford Academic’, *The Journal of Nutrition*, 133(5), p. 1732S–1736S,. doi: 0.1093/jn/133.5.1732S.

Johansson K, Hutcheon JA, Bodnar LM, Cnattingius S, and S. O. (2018) ‘Pregnancy weight gain by gestational age and stillbirth: a population‐based cohort study.’, *An International Journal of Obstetrics & Gynaecology .* doi: doi.org/10.1111/1471-0528.15112.

Johnson-welch, C. *et al.* (2000) *Institutions , Gender and Integrated Approaches.* Davis CA, USA: Broadening Access and Strengthening Input Market Systems.

Kastro S, D. T. and Y. B. (2018) ‘Low birth weight among term newborns in Wolaita Sodo town, South Ethiopia.’, *BMC Pregnancy and Childbirth .*, (18), p. 60. doi: 10.1186/s12884-018-1789-y.

Kennedy, E. *et al.* (2018) ‘Impact of Social and Behavior Change Communication in Nutrition Sensitive Interventions on Selected Indicators of Nutritional Status’, *Journal of human nutrition ·*, 2(1), pp. 24–33.

Kibre K T, Chojenta C, Ellie G, T. G. K. and L. D. (2018) ‘Maternal dietary patterns and risk of adverse pregnancy (hypertensive disorders of pregnancy and gestational diabetes mellitus) and birth (preterm birth and low birth weight) outcomes. a systematic review and meta-analysis .’, *Public Health Nutrition.* doi: 10.1017/S1368980018002616.

Koppmair, S., Kassie, M. and Qaim, M. (2017) ‘Farm production, market access and dietary diversity in Malawi’, *Public Health Nutrition*, 20(2), pp. 325–335. doi: 10.1017/S1368980016002135.

Kuchenbecker, Judith; Reinbott, Anika; Mtimuni, B. et al. (2017) ‘Nutrition education improves dietary diversity of children 6-23 months at community-level :Results from a cluster randomized controlled trial in Malawi.’, *PLoS ONE*, 12(4), p. e0175216 (ISSN: 1932-6203). doi: 10.1371/​journal.pone.0175216.

Kumar N, Harris J, A. R. R. (2015) ‘If They Grow It, Will They Eat and Grow_ Evidence from Zambia on Agricultural Diversity and Child Undernutritio.’, *The Journal of Development Studies*, 51(8). doi: 10.1080/00220388.2015.1018901.

Kumar N, Phuong P H, Harris J, D Harvey, R. R. &Ruel M. T. (2018) ‘What it takes : evidence from a nutrition- and gender-sensitive agriculture intervention in rural Zambia’, *Journal of Development Effectiveness*. Routledge, 10(3), pp. 341–372. doi: 10.1080/19439342.2018.1478874.

Longo S, Bollani L, Decembrino L, Di Comite A, A. M. & S. M. (2013) ‘Short-term and long-term sequelae in intrauterine growth retardation (IUGR).’, *Maternal-Fetal & Neonatal Medicine*, 26(3), pp. 222–225. doi: https://doi.org/10.3109/14767058.2012.715006.

Maine, D. (2000) ‘Role of nutrition in the prevention of toxemia’, *American Journal of Clinical Nutrition*. doi: 10.1093/ajcn/72.1.298S.

Mariyam, A. F. and Dibaba, B. (2018) ‘Epidemiology of Malnutrition among Pregnant Women and Associated Factors in Central Refit Valley of Ethiopia.’, *Journal of Nutritional Disorders & Therapy*, 8(1), pp. 1–8. doi: 10.4172/2161-0509.1000222.

Masset E, Haddad L, C. A. and I. J. (2012) ‘Effectiveness of agricultural interventions that aim to improve nutritional status of children: systematic review.’, *THE BMJ*, 344, p. d8222. doi: https://doi.org/10.1136/bmj.d8222.

Milad Azami, Tayebe Azadi, Sepidezahra Farhang, Shoobo Rahmati, and K. P. (2017) ‘The effects of multi mineral-vitamin D and vitamins (C+E) supplementation in the prevention of preeclampsia.’, *Int J Reprod Biomed (Yazd).*, 15(5), pp. 273–278.

Nana A and Tona Z. (2018) ‘Dietary practices and associated factors during pregnancy in northwestern Ethiopia .’, *BMC Pregnancy and Childbirth*, 183. doi: 10.1186/s12884-018-1822-1.

Nyaruhucha CN, Msuya JM, Noowi B, and G. D. (2006) ‘Maternal weight gain in second and third trimesters and their relationship with birth weights in Morogoro Municipality, Tanzania’, *Tanzania health research bulletin*, pp. 41–44. doi: 10.4314/thrb.v8i1.14270.

Olney D K, Bliznashka B, Becquey E, Birba O, A. R. M. (2017) ‘Adding a Water, Sanitation and Hygiene Intervention and a Lipid-Based Nutrient Supplement to an Integrated Agriculture and Nutrition Program Improved the Nutritional Status of Young Burkinabé Children .’, *The FASEB Journal.*, (455.1).

de onis M, dewey K G, Brghi E, Onyango AW, B. M. and D. B. et al. (2013) ‘The World Health Organization’s global target for reducing childhood stunting by 2025_ rationale and proposed actions’, *Maternal &amp; Child Nutrition - Wiley Online Library*, 9(2), pp. 6–26.

de Onis M and Branca F. and . (2016) ‘Childhood stunting: A global perspective’, *Maternal and Child Nutrition*, pp. 12–26. doi: 10.1111/mcn.12231.

Osei A,Pandey P and Nielsen J (2017) ‘Combining Home Garden , Poultry , and Nutrition Education Program Targeted to Families With Young Children Improved Anemia Among Children and Anemia and Underweight Among Nonpregnant Women in Nepal’, *Food and Nutrition Bulletin*, 38(1), pp. 49–64. doi: 10.1177/0379572116676427.

Ota, E. *et al.* (2015) ‘Antenatal dietary education and supplementation to increase energy and protein intake .’, *Cochrane Library*. doi: 10.1002/14651858.CD000032.pub3.

Ramalho AA, Martins FA, and K. R. (2017) ‘Food Insecurity during the Gestational Period and Factors Associated with Maternal and Child Health’, *Journal of Nutritional Health & Food Engineering*, 7(4), p. 245. doi: 10.15406/jnhfe.2017.07.00245.

Ramesh D Potdar, Sirazul A Sahariah, Meera Gandhi, et al. (2014) ‘Improving women’s diet quality preconceptionally and during gestation: effects on birth weight and prevalence of low birth weight a randomized controlled efficacy trial in India (Mumbai Maternal Nutrition Project).’, *The American Journal of Clinical Nutrition*, 100(5), pp. 1257–1268. doi: https://doi.org/10.3945/ajcn.114.084921.

Reed C C, Longo M, Chiossi G, Landgren R, C. S. and H. C. W. (2018) ‘Hypertensive Disorders in Pregnancy and Gestational Diabetes: Does Weight Gained in Pregnancy Matter?’, *Obstetrics & Gynecology*, 131, p. 105S. doi: 10.1097/01.AOG.0000533455.45890.87.

Riang RM, Broerse J, and N. A. K. (2017) ‘Food beliefs and practices among the Kalenjin pregnant women in rural Uasin Gishu County, Kenya .’, *Journal of Ethnobiology and Ethnomedicine.*, (13), p. 29.

de Rooij S, Wouters H, Yonker J E, Painter R C, and R. T. J. (2010) ‘Prenatal undernutrition and cognitive function in late adulthood’, *Proc Natl Acad Sci U S A*, 107(37), p. 16881–16886. doi: 10.1073/pnas.1009459107.

Rosen J G, Clermont A, Kodish S R, Mater seck A, Salifou A, Garais R F, and I. S. (2018) ‘Determinants of dietary practices during pregnancy : A longitudinal qualitative study in Niger’, *wiley-Maternal & child nutrition*, pp. 1–10. doi: 10.1111/mcn.12629.

Ruel MT, Alderman H, and the maternal and child nutrition study group. (2013) ‘Maternal and Child Nutrition Nutrition-sensitive interventions and programmes : how can they help to accelerate progress in improving maternal and child nutrition ?’, *The Lancet*. Elsevier Ltd, 6736(13), pp. 1–16. doi: 10.1016/S0140-6736(13)60843-0.

ruel MT, A. H. and the maternal and child nutrition study group. (2013) ‘Nutrition-sensitive interventions and programmes: how can they help to accelerate progress in improving maternal and child nutrition?’, *The Lancet*, 382(9891), pp. 531–551.

Rule M T, Quisumbing A R, A. B. M. (2018) ‘Nutrition-sensitive agriculture : What have we learned so far ?’, *Global Food Security*. Elsevier B.V., 17(September 2017), pp. 128–153. doi: 10.1016/j.gfs.2018.01.002.

Saaka, M. (2012) ‘Maternal Dietary Diversity and Infant Outcome of Pregnant Women in Northern Maternal Dietary Diversity and Infant Outcome of Pregnant Women in Northern Ghana’, *International Journal of Child Health and Nutrition*, 1(2), pp. 148–156. doi: 10.6000/1929-4247.2012.01.02.6.

Sahariah SA, Potdar RD, Gandhi M, Kehoe SH, Brown N, S. H. et al. (2016) ‘A Daily Snack Containing Leafy Green Vegetables, Fruit, and Milk before and during Pregnancy Prevents Gestational Diabetes in a Randomized, Control’, *J Nutr.*, 146(7), p. 1453S–60S. doi: 10.3945/jn.115.223461.

Save the Children (2016) *IMPROVING NUTRITION THROUGH MULTISECTORAL SUPPORT : THE ENGINE*. Addis Ababa,Ethiopia.

Schoenaker, D., Soedamah-Muthu, S. S. and Mishra, and G. D. (2014) ‘The association between dietary factors and gestational hypertension and pre-eclampsia: a systematic review and meta-analysis of observational studies.’, *BMC medicine*, p. 157. doi: 10.1186/s12916-014-0157-7.

Schreinemachers, P., Patalagsa, M. A. and Uddin, N. (2016) ‘Impact and cost-effectiveness of women’s training in home gardening and nutrition in Bangladesh’, *Journal of Development Effectiveness*, pp. 473–488. doi: 10.1080/19439342.2016.1231704.

Shea BJ, Grimshaw JM, Wells GA, et al. (2007) ‘Development of AMSTAR: a measurement tool to assess the methodological quality of systematic reviews.’, *BMC Medical research methodology*. doi: 10.1186/1471-2288-7-10.

de Silva Lopes K, Ota E, Shakya P, et al (2017) ‘Effects of nutrition interventions during pregnancy on low birth weight : an overview of systematic reviews’, *BMJ Glob Health*, 2, p. e000389. doi: 10.1136/bmjgh-2017-000389.

Solomons NW, Vossenaar M, Chomat AM, Doak CM, Koski KG, and S. M. (2015) ‘Stunting at birth_ recognition of early-life linear growth failure in the western highlands of Guatemala’, *Public Health Nutr.*, 18(10), pp. 1737–45. doi: 10.1017/S136898001400264X.

SSchoenaker, D. A. J. M. thu *et al.* (2016) ‘The Role of Energy, Nutrients, Foods, and Dietary Patterns in the Development of Gestational Diabetes Mellitus: A Systematic Review of Observational Studies’, *Diabetes Care*, pp. 16–23. doi: 10.2337/dc15-0540.

UNICEF, WHO and World Bank Group (2017) *LEVELS AND TRENDS IN CHILD MALNUTRITION .* doi: 10.1016/S0266-6138(96)90067-4.

USAID (2018) *Ethiopia : Nutrition Profile*.

Uzma Eram, Tamanna, Z., and Humaira, J. T. (2016) ‘R esearch A rticle TABOOS AND MISCONCEPTIONS ASSOCIATED WITH PREGNANCY AMONG RURALWOMEN IN ALIGARH.’, *International Journal of Information Research and Review.*, 3(12), pp. 3407–3409.

Vieira L, Amorosa J, Matthews K, Bianco A, S. J. and F. S. (2017) ‘Is maternal weight gain during pregnancy associated with fetal growth patterns between the 2nd and 3rd trimester as determined by ultrasound?’, *American Journal of Obstetrics and Gynecology*, 216(1), pp. S71–S72. doi: https://doi.org/10.1016/j.ajog.2016.11.990.

Villar J, Abdel-Aleem H, Meriadi M, Mathai M, Ali MM., Zavaleta N.et al, W. caalcium supplementation for the prevention of preeclampsia trial group (2006) ‘World Health Organization randomized trial of calcium supplementation among low calcium intake pregnant women’, *American Journal of Obstetrics and Gynecology*, 194(3), pp. 639–49. doi: 10.1016/j.ajog.2006.01.068.

Watchs T D, Georgieff M, Cusick S, A. M. B. (2014) ‘Issues in the timing of integrated early interventions.’, *Ann N Y Acad Sci.*, (1308), p. 89–106. doi: 10.1111/nyas.12314.

WHO/UNICEF/WFP (2014) *Global nutrition targets 2025: wasting policy brief (WHO/NMH/NHD/14.8).* Geneva.

Zepro, N. B. (2015) ‘Food Taboos and Misconceptions Among Pregnant Women of Shashemene District, Ethiopia.’, *Science Journal of Public Health*, p. 410. doi: 10.11648/j.sjph.20150303.27.

Zerfu T A, Umeta M, and B. K. (2016) ‘Dietary diversity during pregnancy is associated with reduced risk of maternal anemia, preterm delivery, and low birth weight in a prospective cohort study in rural Ethiopia’, *The American Journal of Clinical Nutrition*, 103(6), pp. 1482–1488. doi: https://doi.org/10.3945/ajcn.115.116798.

Zerfu T A, U. M. and B. K. (2016) ‘Dietary habits, food taboos, and perceptions towards weight gain during pregnancy in Arsi, rural central Ethiopia_ a qualitative cross-sectional study.’, *Journal of Health, Population and Nutrition*, (22), p. 35. doi: doi.org/10.1186/s41043-016-0059-8.

Bhutta, Z. A., J. K. Das, A. Rizvi, M. F. Gaffey, N. Walker, S. Horton, P. Webb, A. Lartey, R. E. Black and T. L. N. I. R. Group (2013). "Evidence-based interventions for improvement of maternal and child nutrition: what can be done and at what cost?" The lancet **382**(9890): 452-477.

Daba, G., F. Beyene, H. Fekadu and W. Garoma (2013). "Assessment of knowledge of pregnant mothers on maternal nutrition and associated factors in Guto Gida Woreda, East Wollega Zone, Ethiopia." Journal of Nutrition & Food Sciences **3**(6): 1.

Diddana, T. Z., G. N. Kelkay, A. N. Dola and A. A. Sadore (2018). "Effect of nutrition education based on health belief model on nutritional knowledge and dietary practice of pregnant women in Dessie Town, Northeast Ethiopia: A cluster randomized control trial." Journal of Nutrition and Metabolism **2018**.

FAO (2018). The state of food security and nutrition in the world 2018: building climate resilience for food security and nutrition, Food & Agriculture Org.

Kedir, H., Y. Berhane and A. Worku (2013). "Khat chewing and restrictive dietary behaviors are associated with anemia among pregnant women in high prevalence rural communities in eastern Ethiopia." PloS one **8**(11): e78601.

Kennedy, E., J. Stickland, M. Kershaw and S. Biadgilign (2018). "Impact of social and behavior change communication in nutrition specific interventions on selected indicators of nutritional status." J Hum Nutr **2**(1): 34-46.

Khalid, H., S. Gill and A. M. Fox (2019). "Global aid for nutrition-specific and nutrition-sensitive interventions and proportion of stunted children across low-and middle-income countries: does aid matter?" Health policy and planning **34**(Supplement_2): ii18-ii27.

Moges, M., A. Worku and E. Loha (2015). "Nutritional Status and Associated Factors among Pregnant Women in Boricha Woreda, Sidama Zone, Southern Ethiopia."

Ruel, M. T., H. Alderman, Maternal and C. N. S. Group (2013). "Nutrition-sensitive interventions and programmes: how can they help to accelerate progress in improving maternal and child nutrition?" The lancet **382**(9891): 536-551.

Seferidi, P., T. Hone, A. C. Duran, A. Bernabe-Ortiz and C. Millett (2022). "Global inequalities in the double burden of malnutrition and associations with globalisation: a multilevel analysis of Demographic and Health Surveys from 55 low-income and middle-income countries, 1992–2018." The Lancet Global Health **10**(4): e482-e490.

UNICEF (2020). "Nutrition, for every child."

Workneh, F., M. Eglovitch, T. Shiferaw, F. Shiferie, H. Amanuel, A. W. Tadesse, A. Worku, S. Isanaka, A. C. Lee and Y. Berhane (2021). "Dietary practices among pregnant women in rural Amhara, Ethiopia." Current Developments in Nutrition **5**(Supplement_2): 698-698.

Zelalem, A., M. Endeshaw, M. Ayenew, S. Shiferaw and R. Yirgu (2017). "Effect of nutrition education on pregnancy specific nutrition knowledge and healthy dietary practice among pregnant women in Addis Ababa." Clinics in Mother and Child Health **14**(3): 265.
