# Supplementary material for: Effects of nutrition education and home gardening interventions on feto-maternal outcomes among pregnant women in Jimma Zone, Southwest Ethiopia: A cluster randomized controlled trial
Source: PLoS One. 2023 Oct 20;18(10):e0288150. doi: 10.1371/journal.pone.0288150 (PMC10588865; doi:10.1371/journal.pone.0288150)
Supplement: Attachment — Submitted filename: renamed_8c51e.docx [file pone.0288150.s010.docx]

# COMMENTS – Manuscript PONE-D-22-11496

Title: “Effects of nutrition education and home gardening interventions on feto-maternal outcomes among pregnant women in Jimma Zone, Southwest Ethiopia: a cluster randomized controlled trial”

**Important note: This review pertains only to ‘statistical aspects’ of the study and so ‘clinical aspects’ [like medical importance, relevance of the study, ‘clinical significance and implication(s)’ of the whole study, etc.] are to be evaluated [should be assessed] separately/independently. Further please note that any ‘statistical review’ is generally done under the assumption that (such) study specific methodological [as well as execution] issues are perfectly taken care of by the investigator(s). This review is not an exception to that and so does not cover clinical aspects {however, seldom comments are made only if those issues are intimately / scientifically related & intermingle with ‘statistical aspects’ of the study}. Agreed that ‘statistical methods’ are used as just tools here, however, they are vital part of methodology [and so should be given due importance].**

**COMMENTS:** Please mention all about the ‘cluster(s)’ [like cluster size, how many clusters in population, etc, etc. very briefly] in the ABTRACT [or Methods at least]. In ‘Methods’ you have just said that “A total of 348 pregnant women were recruited at the baseline and 336 attended the end-line survey” which does not define the ‘cluster(s)’. Although in lines 104-5 it is stated that “Then, non-adjacent clusters or kebeles (the smallest administrative units) were selected following the mapping of clusters by geographic information system (GIS) expertise to have buffer zones”, this may not suffice. Later in lines 109-111 you stated that “After stratifying the randomization sequence by the districts using permuted balanced block randomization (block size 18), 6 clusters were assigned to each study arm by the principal investigator” which is confusing. How do/did you stratified the randomization sequence by the districts using permuted balanced block randomization? Basically, ‘do we stratify the randomization sequence?’ is also a question.

Is/Are the cluster(s) size same? {cluster size=112/6=18.67, according to table-2 sample size of each group is 112 [or 116 according to lines 96-7: (116 per each of the three study arms)] then cluster size=116/6=19.33 & Number of clusters in each group are 6}. Is there anything wrong? How otherwise the cluster size is in fraction? You specified the block size as 18 but the number of clusters selected for each group are 6. Is not that confusing? In fact, whenever we use ‘Permuted Block Randomization’ we generally do not reveal the block size. And we generally select the block size randomly to avoid possible/easy prediction of allocation. You may know that the block size is in multiples of number of arms but it is never fixed [in advance] as we choose the block size randomly. Note that it is difficult to manage the block size as large as 18.

You may be absolutely right, in all these, however, remember that this is a scientific/academic document and so all details should be clearly/correctly communicated.

You must have tested ‘Median age(years)’ by Kruskal-Wallis’ test in Table-2. If ‘yes’, you are supposed to mention that [name of (if) another test used] in foot-note, I guess. To provide a description of baseline characteristics is entirely reasonable (since it is clearly important in assessing to whom the results of the trial can be applied), however, statistical comparison of baseline characteristics [last ‘p-value’ column in Table 2] is not desirable at all [because even if P-value turns out to be significant (while comparing baseline characteristics despite random allocation), it is, by definition, a false positive] as you then are supposed to be testing ‘randomization’ then, which in any single trial may not balance all baseline characteristics because ‘randomization’ is a sort of ‘insurance’ and not a guarantee scheme.

References:

1.	Stuart J. Pocock, et al., ‘Subgroup analysis, covariate adjustment and baseline comparisons in clinical trial reporting: current practice and problems’, Statistics in medicine, 2002; 21:2917–2930 [Particularly page 2927]

2.	Harrington D, et al., ‘New guidelines for statistical reporting in the journal’, N Engl J Med 2019;381:285-6

[Important message (indirectly/ultimately indicated) from these articles: Never do any comparison with respect to ‘baseline’ characteristics {by applying statistical significance test(s)}, when allocation is done randomly].

Please note that any regression techniques are not basically/originally developed for any sort of [between or within group(s)] comparison(s) including Generalized estimating equation analysis (GEE). Refer to lines 281-2: “The difference in feto-maternal outcomes (birth weight and hemoglobin level) between the intervention and control arms was examined using a linear GEE”. Otherwise using GEE is very good.

Though the measures/tools used are appropriate [examples, the Pittsburgh sleep quality index (PSQI), Household food insecurity access scale (HHFIAS), the knowledge-attitude-practice (KAP) scores, Minimum dietary diversity – women (MDD-W) score], they {few of them} may yield data that are in [at the most] ‘ordinal’ level of measurement [and not in ratio level of measurement for sure {as the score two times higher does not indicate presence of that parameter/phenomenon as double (for example, a Visual Analogue Scales VAS score or say ‘depression’ score)}]. Then application of suitable non-parametric test(s) is/are indicated/advisable [even if distribution may be ‘Gaussian’ (also called ‘normal’)]. Agreed that there is/are no non-parametric test(s)/technique(s) available to be used as alternative in all situation(s) [suitable / most desired/applicable], but should be used whenever/wherever they are available [note that generalized estimating equation analysis (GEE) is/are parametric].

I request authors to briefly explain what you mean by (line 106) “The study clusters in the area were generated using ArcGIS version 10.3” [because we generally deal with naturally formed clusters]. Although I do not completely agree with all ‘the strengths of this study’ enumerated/listed (lines 483 onwards), I definitely/confidently say that this study and the manuscript are excellent except few points highlighted above. Therefore, recommending minor revision.
