# Supplementary material for: Effects of nutrition education and home gardening interventions on feto-maternal outcomes among pregnant women in Jimma Zone, Southwest Ethiopia: A cluster randomized controlled trial
Source: PLoS One. 2023 Oct 20;18(10):e0288150. doi: 10.1371/journal.pone.0288150 (PMC10588865; doi:10.1371/journal.pone.0288150)
Supplement: Attachment — Submitted filename: Response to reviewers.docx [file pone.0288150.s011.docx]

**Manuscript ID:** PONE-D-22-11496

**Title of Manuscript:** Effects of nutrition education and home gardening interventions on feto-maternal outcomes among pregnant women in Jimma Zone, Southwest Ethiopia: a cluster randomized controlled trial

**Dear Editor,**

We would like to say thank you for assigning a reviewer and returning the crucial comments and recommendations. We found the points raised by the editor and reviewer very interesting to make this article scientifically sound. We addressed all the comments raised by the editor as well as reviewers and wrote clarifications here below for mutual understanding.

**Editor(s)' /Reviewers/ comments and authors' response**

| 1. Editor(s)' Comments | |
| --- | --- |
| **Comments and Recommendations** | **Authors Response** |
| 1. Please ensure that your manuscript meets PLOS ONE's style requirements, including those for file naming. | Thank you for your comment. As per the comment given, we have revised the current document to meet the styles and requirements of Plos One. |
| 2. We note that in your manuscript you state that you recruited underage women (less than 18 years of age), i.e., girls, in your study. Please clarify how many girls participated in your study, and please amend your language to reflect that underage women should be referred to as 'girls' | Thank you for the concern and important comments. We have amended the language in the current document. Page 13 # line 294-295 and page 15 # line 277-278.  39 girls (15.2%) participated in the study. |
| 3. We note that you have indicated that data from this study are available upon request. PLOS only allows data to be available upon request if there are legal or 4. ethical restrictions on sharing data publicly | Again thank you for your comments. As per the comment given we upload the data as S1. No legal/ ethical restrictions. |
| 5. We note that Figure 2 in your submission contains [map/satellite] images which may be copyrighted | Thank you once again for your valuable comments and concerns. We confirm that, we did not utilized any external data to create the figure. Likewise, we did not obtained any base maps or shapes from anywhere as well. The map was solely produced by the authors using ArcGIS 10.3 version software. The database used for preparation was **Ethio-GIS (Ethiopian Geographical Information System). 2004**. A Geo-referenced Database and Digital Terrain Model of Ethiopian Spatial Information Prepared in a Geographical Information System (GIS) Database. Berne, Switzerland: CDE (Centre for Development and Environment), University of Berne. We have put the source on the current revised document. Page # 6 line, 98. |
| 6. Please include a copy of Table 1 which you refer to in your text on page 8.   . | Thank you for your comment. We have included it in the current revised document. # Table 1 |
| **Reviewer comments** | |
| 1. Please mention all about the ‘cluster(s)’ [like cluster size, how many clusters in population, etc, etc. very briefly] in the ABTRACT [or Methods at least]. | Thank you very much for your comments. As per the comment, a revision was made in the current document on the methods part. Page 6# lines 100-114. |
| 2. Later in lines 109-111 you stated that “After stratifying the randomization sequence by the districts using permuted balanced block randomization (block size 18), 6 clusters were assigned to each study arm by the principal investigator” which is confusing. | Thank you for your valuable critical comments. We found the comment is very interesting and of great significance to enrich our manuscript. So, as per the comments given, we have revised it in the current document and made it clear. Page 6# lines 100-103. |
| 3. Is/Are the cluster(s) size the same? {Cluster size=112/6=18.67, according to table-2 sample size of each group is 112 [or 116 according to lines 96-7: (116 per each of the three study arms)] then cluster size=116/6=19.33 & Number of clusters in each group are 6}. Is there anything wrong? | We would like to thank you again. We apologize for the ambiguity made. We have revised and re-write the current document and corrected it. To make it clear we have 18 clusters (8 from Mana district and 10 from Seka chekorsa). Our study arms were three (Husband, Peers, and the controls). Then we randomly assigned clusters (6 for each arms) equally. Finally, all individuals in the clusters fulfilling the criteria were enrolled until the required sample size was met. Therefore, 116 was the number of individuals (1^st Trimester^ pregnant women) enrolled at each study arm on the baseline, and 112 was the number of women we have got at the end-line per the clusters. Page 6 # lines 100-114 |
| 4. You must have tested ‘Median age (years)’ by Kruskal-Wallis test in Table-2. If ‘yes’, you are supposed to mention that [name of (if) another test used] in foot-note. | Thank you so much. It was mentioned in the footnote of table 2 in the current revised document. |
| 5. Statistical comparison of baseline characteristics [last ‘p-value’ column in Table 2] is not desirable at all [because even if P-value turns out to be significant (while comparing baseline characteristics despite random allocation | Thank you very much for your comments on such an important core issue of the study. As per the comment given, we have omitted it. # Table 2. |
| 6. Regression techniques are not basically/originally developed for any sort of [between or within group(s)] comparison(s) including Generalized estimating equation analysis (GEE). Refer to lines 281-2: “The difference in feto-maternal outcomes (birth weight and hemoglobin level) between the intervention and control arms | Thank you for your critical comments. In the current document, we revised and corrected it. Page # 13, lines 282-284 |
| 7. Though the measures/tools used are appropriate [examples, the Pittsburgh sleep quality index (PSQI), Household food insecurity access scale (HHFIAS), the knowledge-attitude-practice (KAP) scores, Minimum dietary diversity – women (MDD-W) score], they {few of them} may yield data that are in [at the most] ‘ordinal’ level of measurement [and not in ratio level of measurement for sure {as the score two times higher does not indicate presence of that parameter/phenomenon as double (for example, a Visual Analogue Scales VAS score or say ‘depression’ score)}]. Then application of suitable non-parametric test(s) is/are indicated/advisable [even if distribution may be ‘Gaussian’ (also called ‘normal’)]. | Thank you very much again. As per the recommendation given, we have re-analyzed the data using the non-parametric (Kruskal Walis) method and presented the nutritional Knowledge, attitude, practice, and Minimum dietary diversity – women (MDD-W) score variables to compare the value difference across the study arms (Page 17 #line 307-230). Regarding the Pittsburgh sleep quality index (PSQI) and Household food insecurity access scale (HHFIAS) variable we have computed only to observe their association with the primary outcomes in GEE. |
| 8. I request authors to briefly explain what you mean by (line 106) “The study clusters in the area were generated using ArcGIS version 10.3” [because we generally deal with naturally formed clusters]. | Thank you for your comments. Sorry for the ambiguity made.  As the editor mentioned the clusters were not made by us, they were naturally formed clusters. What we want to mention is to explain that we have used ArcGIS version 10.3 software to do the study map. In the current document, we have revised it. Page 6 # line 98. |
| 9. I do not completely agree with all ‘the strengths of this study’ enumerated/listed (lines 483 onwards). | Again thank you very much. We appreciate the critical observation of the reviewer. As per the comment given, we have revised the strength of this study in the current document. Page 25 # line 461-467 |

**Thank you in advance!**

Melesse Niguse Kuma

Corresponding author
