# Supplementary material for: Effects of nutrition education and home gardening interventions on feto-maternal outcomes among pregnant women in Jimma Zone, Southwest Ethiopia: A cluster randomized controlled trial
Source: PLoS One. 2023 Oct 20;18(10):e0288150. doi: 10.1371/journal.pone.0288150 (PMC10588865; doi:10.1371/journal.pone.0288150)
Supplement: Attachment — Submitted filename: Response to reviewers.docx [file pone.0288150.s012.docx]

**Manuscript ID:** PONE-D-22-11496

**Title of Manuscript:** Effects of nutrition education and home gardening interventions on feto-maternal outcomes among pregnant women in Jimma Zone, Southwest Ethiopia: a cluster randomized controlled trial

**Dear Editor,**

We would like to say thank you for assigning a reviewer and returning the crucial comments and recommendations. We found the points raised by the editor and reviewer very interesting to make this article scientifically sound. We addressed all the comments raised by the editor as well as reviewers and wrote clarifications here below for mutual understanding.

**Editor(s)' /Reviewers/ comments and authors' response**

| 1. Editor(s)' Comments | |
| --- | --- |
| **Comments and Recommendations** | **Authors Response** |
| 1. You present Difference-In-Differences estimates, but the mehtods for this were not stated in the Data Analysis section. How did you get your estimates/CIs/p-values for these? These are your most important results! Which leads me to my following point. | Thank you for your comments. As per the comment given, we have revised the current document. Page 13# line 281-90 |
| 2. Your GEE models don't seem to show DID estimates, but only main effects. I think it is crucial to get DID estimates from models, as you recognize there were very important group differences at baseline that may bias your results. The way to get DID estimates from regression models is to include an interaction term of time(baseline/endline)*treatment interaction. It seems you have not done this. I insist, this is the most important result of your work so please go through this carefully | Thank you for the concerns and important comments. We have regressed the GEE analyses again and included them in the current document as per the comment given. Page 18 # 331-344 and Table 5. |
| 3. Minor issue: please use g instead of gm or gms in accordance with the International System of Units. | Again thank you for your comments. As per the comment given we have revised the entire document and corrected the current manuscript. |
| **Reviewer comments** | |
| 1. Have not understood statements like (‘what exactly is the meaning of) Foot-note of table-2 which says “Footnote: the median age value is from kruskal wallis?]. Moreover use of ‘English’ language is poor, making the presentation unclear/unimpressive | Thank you very much for your comments. Sorry for the unclarity made during editing. As per the comment, a revision was made in the current document in the footnote of table 2. |
| 2. I am not very much impressed about the study & manuscript [as there are many unanswered questions regarding ‘cluster’ (size, formation/constitution, etc.), | Thank you for your valuable comments. We found the comment has great significance to enrich our manuscript. So, as per the comments given, we have revised it in the current document and made it clear. Page 6 -5# lines 102-108. |

**Thank you in advance!**

Melesse Niguse Kuma

Corresponding author
