# Supplementary material for: Effects of nutrition education and home gardening interventions on feto-maternal outcomes among pregnant women in Jimma Zone, Southwest Ethiopia: A cluster randomized controlled trial
Source: PLoS One. 2023 Oct 20;18(10):e0288150. doi: 10.1371/journal.pone.0288150 (PMC10588865; doi:10.1371/journal.pone.0288150)
Supplement: Attachment — Submitted filename: Response to reviewers.docx [file pone.0288150.s013.docx]

**Manuscript ID:** PONE-D-22-11496

**Title of Manuscript:** Effects of nutrition education and home gardening interventions on feto-maternal outcomes among pregnant women in Jimma Zone, Southwest Ethiopia: a cluster randomized controlled trial

**Dear Editor,**

We would like to say thank you for assigning a reviewer and returning the crucial comments. We found the points raised by the editor and reviewer very interesting to make this article scientifically sound. We addressed all the comments raised by the editor as well as reviewers and wrote clarifications here below for mutual understanding.

**Editor(s)' /Reviewers/ comments and authors' response**

| 1. Editor(s)' Comments | |
| --- | --- |
| **Comments and Recommendations** | **Authors Response** |
| 1. please keep Table 5 | Thank you for your comments. As per the comment given, we have kept Table 5 in the current revised manuscript |
| 2 Drop tables 6 and 7 as they are redundant | Thank you for the concerns and important comments. We have removed Tables 6 and 7 from the revised current document. |
| 3 Provide the full set of estimates of models presented in Table 5 in supplementary tables | Thank you. We have provided the full set of models presented in Table 5 as Supplements(4-9) |
| 4 Adjust your results and discussion accordingly | Again thank you for your comments. As per the comment given we have revised the entire document and corrected the current revised manuscript. |
| **Reviewer comments** | |
| 1. Divided your manuscript into small sections like ‘Objective(s)’, ‘Methods’, ‘Results’, ‘Conclusions | Thank you very much for your comments. As per the comment, a revision was made to the current document. |
| 2. ‘What exactly do you by “Equal allocations were given to each cluster” in line 116? | Thank you for your comments. Sorry for the confusion made during the edition. So, as per the comments given, we have revised it in the current document. It is to mention that each of the study arms has an equal number of pregnant women. |
| 3. Why used the term “Moreover,” in lines 131-32? | Again thank you for your comments. We have corrected in the revised current document. |
| 4. In line 157 you say “This study is not designed to test any one specific theoretical model”.Why? | Thank you very much for your valuable comments. We have corrected the current revised document as per the comment given. |

**Thank you in advance!**

Melesse Niguse Kuma

Corresponding author
