# Supplementary material for: Effects of nutrition education and home gardening interventions on feto-maternal outcomes among pregnant women in Jimma Zone, Southwest Ethiopia: A cluster randomized controlled trial
Source: PLoS One. 2023 Oct 20;18(10):e0288150. doi: 10.1371/journal.pone.0288150 (PMC10588865; doi:10.1371/journal.pone.0288150)
Supplement: Attachment — Submitted filename: Response to reviewers.docx [file pone.0288150.s014.docx]

**Manuscript ID:** PONE-D-22-11496

**Title of Manuscript:** Effects of nutrition education and home gardening interventions on feto-maternal outcomes among pregnant women in Jimma Zone, Southwest Ethiopia: a cluster randomized controlled trial

**Dear Editor,**

We would like to say thank you for returning the document with crucial comments. We found the points raised were very interesting to make this article scientifically sound. We addressed all the comments raised and wrote clarifications here below for mutual understanding.

**Editor(s)' /Reviewers/ comments and authors' response**

| 1. Editor(s)' Comments | |
| --- | --- |
| **Comments and Recommendations** | **Authors Response** |
| 1. In the abstract: please briefly define the three intervention arms in the methods section | Thank you for your comments. As per the comment given, we revised the current manuscript. # page 2 lines 19-22 |
| 2 In the abstract: please clarify that your effect estimates were obtained through a Difference-in-Differences approach | Thank you very much again. We have removed in the revised current document.#page 2 lines 28 &29 |
| 3 State in your description of the GEE statistical models in the main text, that interaction terms of the (intervention group)*(time) were included in order to obtain Difference-in-Differences effect estimates. | Thank you. We have included this in the revised current document.#page 18 lines 306 &307 |
| 4 Once again: please avoid using the term "non-parametric variables" as it is inaccurate. | Again thank you for your comments. As per the comment given we have revised the current manuscript. # page17 line 295 & 296. |
| 5 Please check overall grammar, punctuation, and capitalization. | Thank you very much for your comments. As per the comment given revision was made to the current document and corrected as possible. |

**Thank you in advance!**

Melesse Niguse Kuma

Corresponding author
